# Contributions of the RhoA guanine nucleotide exchange factor *Net1* to polyoma middle T antigen-mediated mammary gland tumorigenesis and metastasis

**DOI:** 10.1186/s13058-018-0966-2

**Published:** 2018-05-16

**Authors:** Yan Zuo, Arzu Ulu, Jeffrey T. Chang, Jeffrey A. Frost

**Affiliations:** 1 Department of Integrative Biology and Pharmacology, University of Texas Health Science Center at Houston, 6431 Fannin St, Houston, TX 77030 USA; 2 School of Biomedical Informatics, University of Texas Health Science Center at Houston, 6431 Fannin St, Houston, TX 77030 USA

**Keywords:** RhoA, Net1, Polyoma middle T antigen, Breast cancer, Metastasis

## Abstract

**Background:**

The RhoA activating protein Net1 contributes to breast cancer cell proliferation, motility, and invasion in vitro, yet little is known about its roles in mammary gland tumorigenesis and metastasis.

**Methods:**

*Net1* knockout (KO) mice were bred to mice with mammary gland specific expression of the polyoma middle T antigen (PyMT) oncogene. Mammary gland tumorigenesis and lung metastasis were monitored. Individual tumors were assessed for proliferation, apoptosis, angiogenesis, RhoA activation, and activation of PyMT-dependent signaling pathways. Primary tumor cells from wild-type and *Net1* KO mice were transplanted into the mammary glands of wild-type, nontumor-bearing mice, and tumor growth and metastasis were assessed. Gene expression in wild-type and *Net1* KO tumors was analyzed by gene ontology enrichment and for relative activation of gene expression signatures indicative of signaling pathways important for breast cancer initiation and progression. A gene expression signature indicative of Net1 function was identified. Human breast cancer gene expression profiles were screened for the presence of a Net1 gene expression signature.

**Results:**

We show that *Net1* makes fundamental contributions to mammary gland tumorigenesis and metastasis. *Net1* deletion delays tumorigenesis and strongly suppresses metastasis in PyMT-expressing mice. Moreover, we observe that loss of *Net1* reduces cancer cell proliferation, inhibits tumor angiogenesis, and promotes tumor cell apoptosis. *Net1* is required for maximal RhoA activation within tumors and for primary tumor cell motility. Furthermore, the ability of PyMT to initiate oncogenic signaling to ERK1/2 and PI3K/Akt1 is inhibited by *Net1* deletion. Primary tumor cell transplantation indicates that the reduction in tumor angiogenesis and lung metastasis observed upon *Net1* deletion are tumor cell autonomous effects. Using a gene expression signature indicative of Net1 activity, we show that Net1 signaling is activated in 10% of human breast cancers, and that this correlates with elevated proliferation and PI3K pathway activity. We also demonstrate that human breast cancer patients with a high *Net1* gene expression signature experience shorter distant metastasis-free survival.

**Conclusions:**

These data indicate that Net1 is required for tumor progression in the PyMT mouse model and suggest that Net1 may contribute to breast cancer progression in humans.

**Electronic supplementary material:**

The online version of this article (10.1186/s13058-018-0966-2) contains supplementary material, which is available to authorized users.

## Background

Metastasis is the primary cause of death in breast cancer patients, yet there are few therapies directed at this process. As regulators of cell proliferation, cytoskeletal organization, and cell motility, Rho GTPases are essential to dissemination of cancer cells throughout the body. The Rho GTPase family consists of 20 genes in humans, with Cdc42, Rac1, and RhoA being the most thoroughly studied [1, 2]. Rac1, RhoA, and RhoC are commonly overexpressed in human breast cancers and RNAi-mediated knockdown or deletion of these genes inhibits tumorigenesis and metastasis [3–7]. Rac1 and RhoA/C contribute to metastasis by regulating separate types of invasive behavior in cancer cells, with Rac1 driving integrin-dependent, mesenchymal-type movement and RhoA/C driving integrin-independent, amoeboid movement. Importantly, cancer cells switch between these types of movement depending on the extracellular obstacles they are traversing, and inhibition of either of these forms of movement significantly inhibits invasive activity [8–10].

Rho GTPases function as molecular switches, cycling between their active, GTP-bound and inactive, GDP-bound states. When active, Rho proteins interact with downstream proteins, known as effectors, to initiate signaling cascades that control cell motility [11, 12]. Rho GTPase activation is controlled by two families of proteins, known as guanine nucleotide exchange factors (RhoGEFs) and GTPase activating proteins (RhoGAPs). RhoGEFs stimulate GDP release, thereby allowing Rho proteins to bind GTP and become active [13, 14]. RhoGAPs stimulate the intrinsic GTPase activity of Rho proteins, thereby shutting them off [15]. Because the wild-type forms of Rho proteins are overexpressed in breast cancers, it is commonly assumed that altered function of RhoGAPs and RhoGEFs drives their activation. For example, the RhoGAP DLC-1 is deleted or epigenetically silenced in many breast cancer subtypes, driving aberrant RhoA activation and metastatic spread to the bones [16–18]. Alternatively, the Rac1 GEFs P-Rex1, Vav2/3, and Dock1 have been shown to contribute to metastatic behavior in particular breast cancer subtypes [19–21]. Despite evidence for RhoA and RhoC contributing to breast cancer tumorigenesis and metastasis, RhoA subfamily GEFs that contribute to breast cancer in vivo have not yet been identified.

The neuroepithelial transforming gene 1 (Net1) is a RhoA subfamily GEF that is overexpressed in many human cancers, including breast cancer [22, 23]. We have shown previously that Net1 is required for human breast cancer cell motility and invasive capacity in vitro [24]. In these cells Net1 is dedicated to controlling actomyosin contraction, as inhibition of Net1 expression blocks actomyosin contractility but does not affect other RhoA-regulated events, such as Ezrin phosphorylation. We have also observed that Net1 controls FAK activation, which is necessary for focal adhesion maturation [24]. Furthermore, Net1 has been shown to control cell motility in other cell types, and to regulate actin cytoskeletal rearrangements downstream of ligands such as TGFβ [25–27]. Net1 also controls mitotic progression by regulating Aurora A activation and chromosome alignment during the metaphase [28]. Thus, there is ample evidence to suggest that Net1 may contribute to tumorigenesis and metastasis in vivo; however, the role of Net1 in these processes has not been investigated. Similarly, the role of Net1 in human breast cancer is largely unknown.

In the present work we demonstrate that *Net1* is critical for mammary gland tumorigenesis and metastasis in the mouse mammary tumor virus (MMTV)-PyMT mouse genetic model of breast cancer, and demonstrate obligate signaling pathways that are regulated by *Net1*. Moreover, we identify a gene expression signature indicative of Net1 function and use this signature to demonstrate that Net1 contributes to metastasis in human breast cancer patients. Together these data indicate that Net1 is required for breast cancer progression in the MMTV-PyMT mouse model and may also contribute to human breast tumorigenesis and metastasis.

## Methods

### Mouse husbandry and care

Mice were housed in the Center for Laboratory Animal Medicine and Care within the Medical School at the University of Texas Health Science Center at Houston, TX, USA. All studies were approved by the Institutional Animal Care and Use Committee (protocol AWC 14-007) and were conducted in accordance with the guidelines of the US Public Health Service Policy for Humane Care and Use of Laboratory Animals.

### Mouse strains used, genotyping, and analysis of tumorigenesis

*Net1*^−/−^ mice in the C57BL/6 strain were as described previously [29]. Male *MMTV-PyMT* mice (Tg(MMTV-PyVT)634Mul) in the FVB/J background were purchased from Jackson Labs. The *MMTV-PyMT* allele was carried by crossing to female FVB/J mice. Mice lacking *Net1* were backcrossed to wild-type FVB/J mice for 10 generations to create a congenic line. Female *Net1*^+/−^ (FVB/J) mice were mated with male *MMTV-PyMT* (FVB/J) mice to derive *Net1*^*+/−*^*,MMTV-PyMT* mice. Female *Net1*^*+/−*^ mice were crossed with *male Net1*^*+/−*^*,MMTV-PyMT* mice to produce a cohort of female littermates with *Net1*^*+/+*^*,MMTV-PyMT* and *Net1*^*+/−*^*,MMTV-PyMT*, and *Net1*^*−/−*^*,MMTV-PyMT* genotypes for tumor studies. Primers for genotyping *Net1*-deficient mice were as follows: forward primer 5GF3, 5′-TGCTATGCTATTGCTGCTT-3′, and reverse primer 3GR1, 5′-AGAACACCACCAAGTAACAA-3′ (amplifies wild-type *Net1*); and forward primer 5GF1, 5′-TTGTTACTTGGTGGTGTTCT-3′ and reverse primer TV3-1R, 5′-AAGTGCTAACCTTCCTGC-3′ (amplifies *Net*^*−/−*^ allele) [29]. The PyMT transgene was identified using previously published primers: forward, 5′-CGGCGGAGCGAGGAACTGAGGAGAG-3′; and reverse, 5′-TCAGAAGACTCGGCAGTCTTAGGCG-3′ [30]. Tumor growth was monitored after weaning. Once palpable tumors had formed, tumor size was measured twice per week using electronic calipers. Tumor volume was calculated using the following equation:

$$ V=\left(\mathrm{Length}\times {\mathrm{Width}}^2\right)/2. $$

Mice were euthanized when the largest tumor reached 2.5 cm^3^.

### Antibodies

The following antibodies were used: anti-pSer19-MLC2 (3675), anti-Ki67 (12202), anti-cleaved caspase 3 (9661), anti-CD31 (77699), anti-pT696-MYPT1 (5163), anti-MYPT1 (2634), anti-pY461-Src (6943), anti-pT202/Y204ERK1/2 (4370), anti-ERK1/2 (4695), anti-pT308-Akt1 (2965), anti-pS473-Akt1 (4060), anti-Akt (2920), anti-PP2A-C (2038), anti-PP2A-A (2041), anti-Shc (2432), anti-PI3 kinase p85 (4257), and anti-RhoC (3430) (Cell Signaling Technology); anti-β-actin (A5316) (Sigma-Aldrich); anti-PyMT (sc-53,481), anti-GST (sc-138), anti-Src (sc-8056), and normal rat IgG (sc-2026) (SantaCruz Biotechnology); and anti-RhoA (ARH03) (Cytoskeleton, Inc.).

### Primary tumor cell isolation and mammary gland transplantation

Individual mammary tumors were isolated from *Net1*^*+/+*^*,MMTV-PyMT* and *Net1*^*−/−*^*,MMTV-PyMT* mice, manually minced, and incubated in DMEM/F12 (Hyclone) with 2 mg/ml collagenase A (Agilent), and 1× antibiotic–antimycotic (Life Technologies) for 2 h at 37 °C with 150 rpm rotation at a 45° angle. The minced tissues were then shaken vigorously and pipetted up and down to create a cell suspension. The cells were pelleted by centrifugation at 600 × *g* for 10 min at room temperature, resuspended in DMEM/F12 with 10% fetal bovine serum (FBS) and 1× antibiotic–antimycotic, and passed through a 70-μm cell strainer (ThermoFisher Scientific). The cells were cultured in DMEM/F12 with 10% FBS, 100 U/ml penicillin/streptomycin, 10 μg/ml insulin, and 1× antibiotic–antimycotic. The epithelial marker cytokeratin 8 (CK8) and PyMT were detected in 90–95% of cells by immunofluorescence microscopy.

Confluent primary tumor cells were harvested with 0.25% trypsin and rinsed twice with PBS. 8 × 10^6^ cells were mixed with Matrigel (catalog no. 354,248; Corning) to a final volume of 200 μl, and were injected into the number four fat pad of wild-type female FVB mice, 6–7 weeks old, using a 28.5-G insulin syringe (catalog no. 329,424; BD). Once palpable, tumor sizes were measured twice per week. Mice were euthanized when the tumors reached 2.5 cm in length or diameter. Mammary tumors and lungs were collected for immunohistochemistry (IHC) and IF staining to analyze tumor cell proliferation, apoptosis, and angiogenesis, as described in the following.

### Cell motility assays

For migration assays, confluent primary tumor cells were starved in DMEM/F12 plus 0.5% FBS for 16 h prior to trypsinization. 8 × 10^4^ cells were placed in the upper chamber of a Transwell insert with 8-μm pores (BD Biosciences). The medium in the bottom well was supplemented with EGF (100 ng/ml; R&D Systems). Cells were allowed to migrate for 2 h, and then the cells in the upper well were removed using a cotton swab. Cells on the bottom of the membrane were fixed and stained with DAPI (1 μg/ml; Sigma-Aldrich). Cells that had traversed the membrane were counted in 10 random fields using a 20× objective and a Zeiss Axiophot microscope. Images were captured with an Axiocam MRm camera and Axiovision software. Cell numbers were quantified using ImageJ software.

### Mammary gland whole mount analysis and tissue immunohistochemistry

After dissection, the fourth inguinal mammary glands were immediately fixed in Carnoy’s fixative (60% ethanol, 30% chloroform, 10% glacial acetic acid) for 2–4 h at room temperature. Glands were stained in Carmine alum solution (2 mg/ml carmine, 10.5 mM aluminum potassium sulfate dodecahydrate) overnight with gentle shaking followed by successive dehydration steps in 70%, 95%, and 100% ethanol for 1 h each, at room temperature. Glands were cleared in xylene overnight and mounted on glass slides with Permount (ThermoFisher Scientific). Mammary glands were imaged with an Eclipse 80i digital camera (Nikon) mounted on a SMZ-745 T stereo microscope (Nikon).

For immunohistochemistry (IHC) of hyperplastic regions, number four inguinal mammary glands were immediately fixed in 4% paraformaldehyde overnight at 4 °C and stored in 70% ethanol at 4 °C until paraffin embedding. Tumors were excised and fixed for IHC as already described. Five-micron sections were cut for all tissues, deparaffinized in xylene, and rehydrated. Sections were boiled for 20 min in 10 mM sodium citrate for antigen retrieval, rinsed in PBS, and quenched for 30 min in 3% H_2_O_2_ at room temperature. Sections were blocked in 5% BSA/0.5% Tween-20, or M.O.M. blocking buffer (BMK2202; Vector Labs), for 1 h at room temperature. Primary antibodies were diluted in blocking solution and sections were incubated with primary antibodies overnight at 4 °C. After washing five times in phosphate buffered saline (PBS), sections were incubated with secondary antibodies for 45 min at room temperature, washed in PBS, and incubated in ABC solution (PK7100; Vector Labs) for 30 min. Sections were then developed in diaminobenzidine (K3468; Dako) and counterstained with hematoxylin (Thermo Fisher Scientific). Images were visualized with Eclipse 80i microscope (Nikon) and Digital Sight DS-VI1 camera (Nikon), and acquired using NIS-Elements Basic Research software (Nikon).

### Lung isolation and analysis

Whole lungs were isolated after euthanasia, rinsed with sterile PBS, and immediately fixed in 4% paraformaldehyde overnight at 4 °C. Lungs were stored in 70% ethanol at 4 °C until paraffin embedding. Metastatic foci on the dorsal and ventral surface of lung lobes were counted and imaged with a SMZ-745 T stereo microscope (Nikon) mounted with an Eclipse 80i digital camera (Nikon). Five-micron sections were cut, deparaffinized in xylene, and rehydrated. The lung sections were then stained with hematoxylin and eosin (ThermoFisher Scientific). Sections were visualized with a SMZ-745 T stereo microscope (Nikon), and images were acquired using NIS-Elements Basic Research software (Nikon).

### Isolation of tissues and western blotting analysis

Mouse tissues were rinsed quickly in cold PBS, snap frozen in liquid nitrogen, and stored at − 80 °C until use. For extraction of proteins and mRNA, frozen tissues were pulverized with a mortar and pestle under liquid nitrogen and homogenized on ice in SDS lysis buffer for protein extraction (2% SDS, 20 mM Tris–HCl (pH 8.0), 100 mM NaCl, 80 mM β-glycerophosphate, 50 mM NaF, 1 mM sodium orthovanadate, 10 μg/ml pepstatin A, 10 μg/ml leupeptin, 10 μg/ml aprotinin) or TRK lysis buffer (E.Z.N.A.® Total RNA Kit I; Omega Bio-Tek) for RNA extraction, using a rotor-stator homogenizer. For protein analysis, lysed tissue was sonicated and protein concentrations were determined by bicinchoninic acid assay (Pierce). Equal amounts of protein were separated by SDS-PAGE, transferred to polyvinylidene difluoride membrane (PVDF), and analyzed by western blotting.

### Immunoprecipitation and GST-RBD assays

For analysis of proteins coprecipitating with PyMT, pulverized mouse tumors were lysed in radioimmunoprecipitation assay (RIPA) buffer with 0.1% SDS (1.0% Triton X-100, 0.1% SDS, 0.5% sodium deoxycholate, 50 mM Tris–HCl (pH 8.0), 150 mM NaCl, 80 mM β-glycerophosphate, 10 μg/ml leupeptin, 10 μg/ml pepstatin A,10 μg/ml aprotinin, 1 mM phenylmethylsulfonyl fluoride), incubated on ice for 10 min, and homogenized using a rotor-stator homogenizer. Insoluble proteins were pelleted by centrifugation (16,000 × *g*, 10 min, 4 °C). Equal amounts of soluble lysate were precleared by incubation for 30 min at 4 °C with 2 μg of normal rat IgG plus Protein G-Sepharose (Rockland Immunochemicals). Clarified lysates were then incubated with 2 μg of normal rat IgG or rat anti-PyMT plus Protein G-Sepharose for 2 h at 4 °C. Immunoprecipitates were washed three times with wash buffer (20 mM Tris–HCl (pH 8.0), 125 mM NaCl, 5 mM MgCl_2_, and 0.5% Triton X-100), resuspended in 2 × Laemmli sample buffer, and resolved by SDS-PAGE. Proteins were transferred to a PVDF membrane and analyzed by western blotting analysis as described previously [31].

For GST-RBD assays, pulverized mouse tumors were lysed in 0.5% Triton lysis buffer plus 10 mM MgCl_2_ (0.5% Triton X-100, 10 mM MgCl_2_, 20 mM Tris–HCl (pH 8.0), 100 mM NaCl, 1 mM EDTA, 50 mM NaF, 80 mM β-glycerophosphate, 10 mM MgCl_2_, 1 mM Na_2_VO_3_, 10 μg/ml leupeptin, 10 μg/ml pepstatin A,10 μg/ml aprotinin, 1 mM phenylmethylsulfonyl fluoride) and homogenized using a rotor-stator homogenizer. Insoluble proteins were pelleted by centrifugation (16,000 × *g*, 10 min, 4 °C). Equal amounts of soluble lysate were incubated with 30 μg of GST-Rhotekin-RBD protein beads (Cytoskeleton, Inc.) for 1 h at 4 °C. Precipitates were washed three times with wash buffer (25 mM Tris–HCl (pH 7.5), 30 mM MgCl_2_, 40 mM NaCl), resuspended in 2 × Laemmli sample buffer, and resolved by SDS-PAGE. Proteins were transferred to a PVDF membrane and analyzed by western blotting.

### Gene expression analysis

Gene expression was analyzed by the UTHealth Quantitative Genomics and Microarray Core Facility using an Illumina mouse WG6 Whole-Genome Gene Expression BeadChip. Gene expression values were estimated with the Illumina GenomeStudio software with background subtraction and quantile normalization. Changes in gene expression were initially identified using a Student’s *t* test, and genes were accepted as differentially expressed if they exhibited at least 1.5-fold change with *P* < 0.05 between the wild-type and *Net1* knockout samples. Gene Ontology category enrichment analysis was performed using GATHER [32]. We scored the activation of gene expression signatures on gene expression profiles as described previously [33]. Briefly, for a gene expression dataset, we centered and normalized each gene to a mean of 0 and standard deviation of 1, and then averaged the expression of each gene in the signature, after taking the additive inverse of the expression values for genes negatively correlated with the pathway. Finally, we created a Net1 gene expression signature using an empirical Bayes approach [34] to find genes differentially expressed in *Net1* knockout mouse tumors with at least 5-fold change and *P* < 0.05. To score this signature on human tumors, we found the human orthologs of the genes in the signature using the Homologene database [35].

### Statistical analysis

Unpaired, two-tailed Student *t* tests were employed for all other statistical tests. *P* < 0.05 was considered significant, as indicated in figure legends. All data are reported as means, and errors are the standard error of the mean. Animal cohort size was chosen based on the work of many other groups using this tumor model and all animals within the cohort were included in the results. No randomization of animals was necessary. No blinding of cohort identity was done.

## Results

### *Net1* is required for tumorigenesis and metastasis in MMTV-PyMT mice

We have shown previously that mice lacking *Net1* are healthy, but experience a short delay in mammary gland development during puberty characterized by reduced estrogen receptor alpha (ERα) expression, reduced proliferation, and less ductal branching. However, these mice are able to nurse their young, indicating that *Net1* is ultimately dispensable for mammary gland function [29]. To determine whether *Net1* is required for mammary gland tumorigenesis or metastasis, we bred mice lacking *Net1* to mice carrying the polyoma middle T antigen under the control of the mouse mammary tumor virus promoter (MMTV-PyMT). The MMTV-PyMT mouse model is an extremely aggressive, well-characterized breast cancer model in which mice develop multifocal mammary tumors at a median age of 6–7 weeks. Moreover, lung metastasis occurs in these mice with 100% penetrance [36]. Importantly, this breast cancer model does not require ERα expression for tumorigenesis or disease progression [37].

**Fig. 1:**
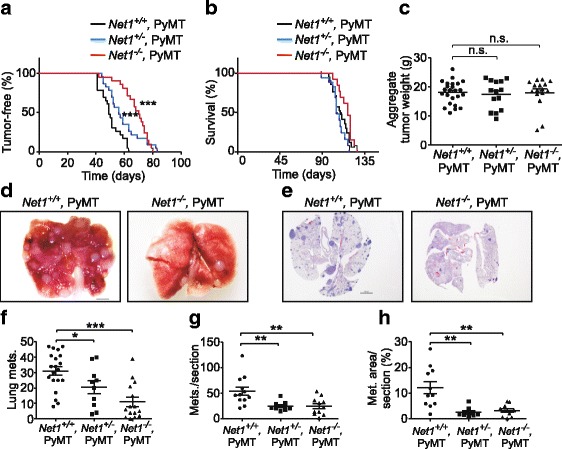
*Net1* deletion delays tumorigenesis and inhibits metastasis in MMTV-PyMT mice. **a** Kaplan–Meier analysis of tumor onset. *Net1*^*+/+*^*,PyMT* = 23 mice; *Net1*^*+/−*^*,PyMT* = 23 mice; *Net1*^*−/−*^*,PyMT* = 21 mice. **b** Survival analysis. Mice were euthanized when the largest tumor reached 2.5 cm. *Net1*^*+/+*^*,PyMT* = 18 mice; *Net1*^*+/−*^*,PyMT* = 14 mice; *Net1*^*−/−*^*,PyMT* = 14 mice. **c** Aggregate tumor mass in MMTV-PyMT mice. *Net1*^*+/+*^ = 22 mice; *Net1*^*+/−*^ = 14 mice; *Net1*^*−/−*^ = 7 mice. **d** Examples of lungs from *Net1*^*+/+*^*,MMTV-PyMT* and *Net1*^*−/−*^*,MMTV-PyMT* mice. Bar = 2 mm. **e** Examples of H&E-stained lung sections from *Net1*^*+/+*^*,MMTV-PyMT* and *Net1*^*−/−*^,*MMTV-PyMT* mice. Bar = 2 mm. **f** Quantification of metastatic nodules on lung surface in genotypes shown. *Net1+/+,PyMT* = 20 mice; *Net1+/−,PyMT* = 10 mice; *Net1−/−,PyMT* = 15 mice. **g** Quantification of metastases in lung sections in genotypes shown. *Net1+/+,PyMT* = 12 mice; *Net1+/−,PyMT* = 9 mice; *Net1−/−,PyMT* = 11 mice. **h** Quantification of lung area occupied by metastases in genotypes shown. *Net1+/+,PyMT* = 11 mice; *Net1+/−,PyMT* = 9 mice; *Net1−/−,PyMT* = 10 mice. Bars represent median values. *P < 0.05; **P < 0.01; ***P < 0.001. Met. metastasis, Mets metastases, Net1 neuroepithelial transforming gene 1, n.s. not significant, PyMT polyoma middle T antigen

To assess the contribution of Net1 to tumorigenesis and metastasis, we bred cohorts of mice lacking one or both *Net1* alleles, and compared the rate of tumor appearance to that of wild-type MMTV-PyMT mice. We observed that loss of one *Net1* allele was sufficient to significantly delay the appearance of palpable tumors, and that loss of both *Net1* alleles caused a more significant effect (Fig. 1a). The effect of deletion of a single *Net1* allele may reflect our prior observation that loss of one allele causes an approximately 70% decrease in *Net1* mRNA expression in the mammary gland [29]. Despite the delay in tumorigenesis, mice lacking *Net1* invariably developed tumors in all mammary glands, with at least one of these tumors reaching maximum allowable size in a time frame similar to wild-type PyMT-expressing mice (Fig. 1b). Moreover, the aggregate tumor weight was similar in all genotypes (Fig. 1c). To determine whether *Net1* deletion affected metastasis, we assessed the lungs of mice within each genotype when mice were euthanized. We observed that loss of *Net1* strongly reduced the number of metastatic nodules observable on the lung surface (Fig. 1d, f). *Net1* deletion also significantly reduced the number of metastases and overall metastatic area in sectioned lungs stained with hematoxylin and eosin (H&E) (Fig. 1e, g, h). These data indicate that *Net1* contributes to mammary gland tumorigenesis and lung metastasis in PyMT-expressing mice.

### Net1 is required for cell proliferation in early lesions

**Fig. 2:**
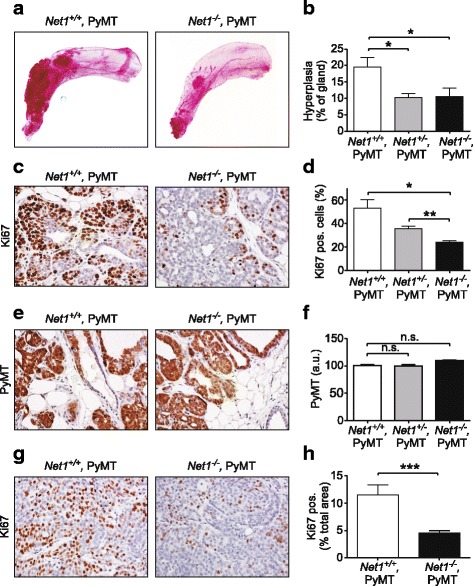
*Net1* deletion delays mammary gland hyperplasia and inhibits proliferation. **a** Representative examples of inguinal mammary gland whole mounts from *Net1*^*+/+*^*,PyMT* and *Net1*^*−/−*^*,PyMT* mice at 5 weeks of age. **b** Quantification of hyperplastic mammary gland area at 5 weeks in genotypes shown. Six mice per genotype analyzed. **c** Representative examples of Ki67 staining in hyperplastic mammary gland sections from mice at 5 weeks. **d** Quantification of Ki67-positive cells in hyperplastic mammary gland sections at 5 weeks. Five independent regions within each sample quantified, 3–4 animals per genotype. **e** Representative examples of PyMT expression in hyperplastic mammary gland sections at 5 weeks. **f** Quantification of PyMT staining in mammary gland sections. Six independent regions within each sample quantified, 3 animals per genotype. **g** Representative examples of Ki67 staining in tumors at 14 weeks. **h** Quantification of Ki67-positive cells in 14-week tumors. Ki67 staining quantified as percent positive area divided by total area. Five independent regions within each sample quantified, 6 animals per genotype. Errors are standard error of the mean. *P < 0.05; **P < 0.01; ***P < 0.001. Net1 neuroepithelial transforming gene 1, n.s. not significant, PyMT polyoma middle T antigen

Hyperplastic areas begin to appear in the ducts surrounding the nipples in MMTV-PyMT mice as early as 3 weeks of age, and by 5 weeks these hyperplastic regions are large enough to easily observe in mammary gland whole mounts [38]. To determine whether *Net1* deletion affected the incidence of hyperplasia, we analyzed mammary gland whole mounts from MMTV-PyMT mice at 5 weeks of age lacking one or both *Net1* alleles. We observed that loss of just one *Net1* allele was sufficient to reduce the overall area of hyperplasia (Fig. 2a, b). This was not due to a general failure of the mammary duct to develop, as the ductal tree had clearly invaded past the lymph node (Fig. 2a, right panel). Ki67 staining indicated that proliferation in these early-stage legions was clearly reduced in mice lacking *Net1* (Fig. 2c, d). This was not due to altered PyMT expression, as *Net1*^*−/−*^mice expressed similar levels of the PyMT transgene (Fig. 2e, f). Proliferation was also significantly reduced in late-stage tumors, indicating that this phenotype was maintained (Fig. 2g, h). These data indicate that *Net1* deletion reduces cell proliferation in early hyperplastic regions and as well as in late-stage tumors.

### *Net1* deletion results in increased tumor necrosis and reduced tumor angiogenesis

**Fig. 3:**
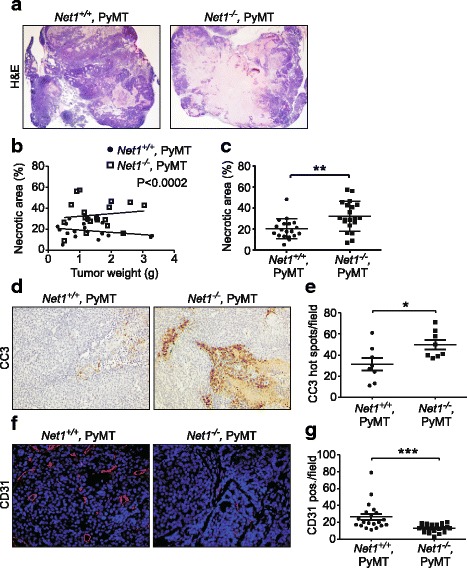
*Net1* deletion inhibits tumor angiogenesis and causes increased tumor cell death. **a** Representative examples of H&E staining of large tumors in genotypes shown. **b** Quantification of necrotic area in H&E sections over range of tumor sizes for genotypes shown. Five animals per genotype, 4–5 tumors per animal, quantified. **c** Quantification of necrotic area of all tumors per genotype. **d** Representative examples of cleaved caspase 3 (CC3) staining in large tumors from genotypes shown. **e** Quantification of number of CC3 hot spots in large tumors. Entire tumor sections analyzed from 8 mice of each genotype. **f** Examples of cell determinant 31 (CD31) staining (red) in solid tumors from *Net1*^*+/+*^*,PyMT* and *Net1*^*−/−*^*,PyMT* mice. DNA shown in blue. **g** Quantification of CD31 staining. Five areas per sample analyzed; tumors from 4 (*Net1*^*+/+*^*,PyMT*) or 5 (*Net1*^*−/−*^*,PyMT*) mice assessed. Bars represent median values. *P < 0.05; **P < 0.01; ***P < 0.001. H&E hematoxylin and eosin, Net1 neuroepithelial transforming gene 1, PyMT polyoma middle T antigen

Most tumors in *Net1*^*−/−*^ mice were less firm than wild-type tumors, suggesting that they contained significant necrotic mass. To determine whether this was the case, we examined the morphology of tumor sections in a range of tumor sizes from less than 0.5 g to greater than 3 g. This analysis indicated that tumors with *Net1* deletion exhibited an increased necrotic area regardless of tumor size (Fig. 3a–c). Necrotic areas were largely confined to the centers of tumors with healthy margins. *Net1* knockout tumors also had an increase in staining for the apoptotic marker cleaved caspase 3 (CC3) (Fig. 3d, e). Because tumor necrosis can be caused by reduced tumor angiogenesis, we examined non-necrotic areas of tumors for expression of the blood vessel endothelial cell marker CD31. This analysis showed that tumors lacking *Net1* had reduced CD31 staining, indicating a reduction in blood vessel content (Fig. 3f, g). These data indicate that loss of *Net1* results in increased tumor necrosis, most likely due to impaired tumor angiogenesis.

### Net1 is required for RhoA signaling in tumors

**Fig. 4:**
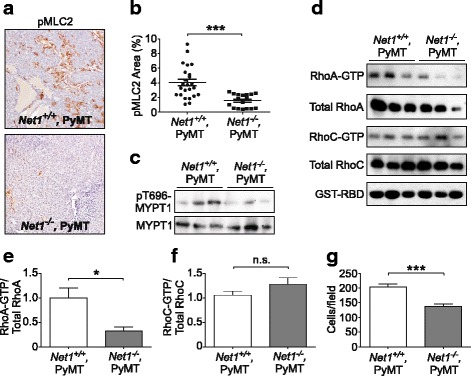
*Net1* deletion inhibits RhoA activation and myosin light chain phosphorylation in tumors. **a** Representative examples of staining for myosin light chain 2 phosphorylated on serine 19 (pMLC2) in genotypes shown. Only solid tumors analyzed. **b** Quantification of pMLC2 staining as percentage of total area in genotypes shown. Seven areas analyzed, 3 animals per genotype. **c** Analysis of myosin phosphatase 1 (MYPT1) phosphorylation on its activating site T696 in tumor lysates. Each lane represents lysate from a tumor from a separate animal. **d** Analysis of active RhoA and RhoC activation in tumor lysates using GST-Rhotekin binding domain (GST-RBD) pulldowns. Each lane represents lysate from a tumor from a separate animal. **e** Quantification of RhoA-GTP/total RhoA for GST-RBD pulldowns. Six tumors per genotype quantified. **f** Quantification of RhoC-GTP/total RhoC for GST-RBD pulldowns. Six tumors per genotype quantified. **g** Quantification of primary tumor cell motility toward EGF in modified Boyden chambers. Data represent mean number of migrated cells/field from three separate tumors per genotype, isolated from separate mice, performed in duplicate. Errors are standard error of the mean. *P < 0.05; ***P < 0.001. GST glutathione-S-transferase, MYPT1 myosin phosphatase targeting subunit 1, Net1 neuroepithelial transforming gene 1, n.s. not significant, PyMT polyoma middle T antigen, RBD RhoA binding domain, RhoA Ras homolog family member A, RhoC Ras homolog family member C

We have observed previously in human breast cancer cells that Net1 controls RhoA activation and actomyosin contractility [24]. Because *Net1*^*−/−*^ tumors exhibited reduced lung metastasis, we assessed RhoA signaling. RhoA controls actomyosin contraction by promoting the accumulation of phosphorylated myosin light chain (MLC2) [1]. When we examined tumors lacking *Net1* for MLC2 phosphorylation we observed that there was a significant decrease in pMLC2 staining (Fig. 4a, b). Western blotting analysis showed that there was also a decrease in phosphorylation of the regulatory subunit of myosin phosphatase (MYPT1) on its activating site pT696 (Fig. 4c). Moreover, there was a significant decrease in the overall level of RhoA activity in these tumors (Fig. 4d, e). However, RhoC activation was unaffected, indicating that Net1 is specific for RhoA in PyMT tumors (Fig. 4d, f). These data indicate that *Net1* deletion caused a substantial reduction in RhoA activity and actomyosin contractility in PyMT-expressing tumors. To determine whether this translated into decreased motility of individual tumor cells, we measured the motility of cells isolated from primary tumors in modified Boyden chambers. We observed that *Net1* deletion significantly impaired motility toward EGF (Fig. 4g), suggesting that these cells would have a reduced capacity for motility in vivo.

### Net1 is required for PyMT signaling

**Fig. 5:**
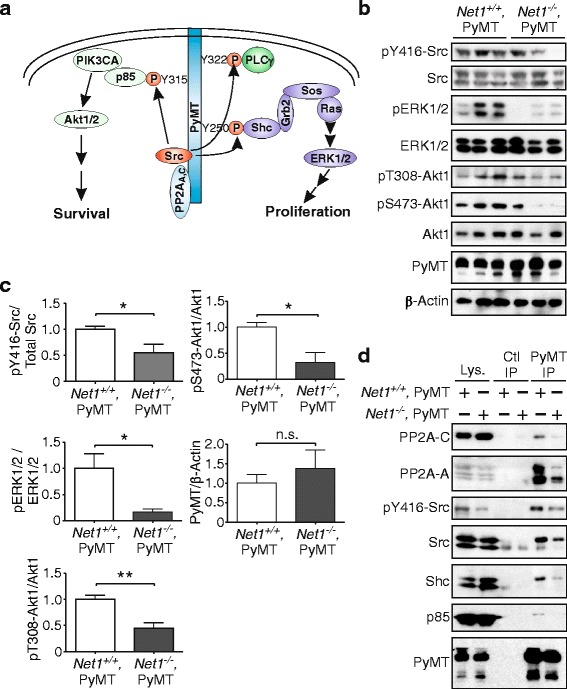
*Net1* deletion inhibits PyMT-dependent ERK1/2 and Akt1 activation. **a** Schematic depicting PyMT-initiated signaling. **b** Representative analysis of Src, ERK1/2, and Akt1 phosphorylation on their activating sites in whole tumor lysates. Each lane represents a tumor from a distinct animal. **c** Quantification of western blotting in whole tumor lysates. Three to 6 tumors per genotype examined. **d** Western blot analysis of proteins coimmunoprecipitating with PyMT from tumors of genotypes shown. Representative experiment from four independent experiments. Errors are standard error of the mean. *P < 0.05; **P < 0.01. AKT1 v-akt murine thymoma viral oncogene homolog 1, ERK1 extracellular signal regulated kinase 1, Net1 neuroepithelial transforming gene 1, p85 phosphatidylinositol 3-kinase regulatory subunit p85α, PIK3CA phosphatidylinositol 3-kinase catalytic subunit A, PP2A protein phosphatase 2A, PyMT polyoma middle T antigen, Shc Src homology domain 2 containing, Src SRC proto-oncogene, non-receptor tyrosine kinase

PyMT is a plasma membrane-associated protein that transforms cells by acting as a scaffold for Shc, the PI3K regulatory subunit p85, and PLCγ (Fig. 5a) [39, 40]. Shc recruitment activates the Ras–Raf–Mek–ERK pathway to promote cell proliferation and tumor growth [41, 42]. p85 recruitment brings the catalytic subunit of PI3K in proximity to the plasma membrane, where it generates phosphatidylinositol (3,4,5)-trisphosphate (PIP_3_) to promote tumor cell survival [41, 42]. Importantly, both of these pathways must be activated for PyMT to promote mammary tumorigenesis [41]. To assess whether *Net1* deletion impaired signaling through these pathways, we examined Src, ERK1/2, and Akt1 activation in wild-type and *Net1* knockout tumors. For these assays we selected healthy, solid sections of tumors from each genotype. We observed a significant decrease in Src activation in *Net1* knockout tumors (Fig. 5b, c). There was also a significant decrease in ERK1/2 activation, as well as phosphorylation of Akt1 on its activating sites T308 and S473 (Fig. 5b, c). These data suggest that the ability of PyMT to stimulate intracellular signaling was impaired by loss of *Net1*.

To initiate cell signaling PyMT must first recruit the A and C subunits of PP2A, which then allows recruitment of Src [43, 44]. Src interaction with PyMT stimulates its tyrosine kinase activity, promoting phosphorylation of residues within PyMT that serve as docking sites for Shc, p85, and PLCγ [39, 40]. To determine whether the interaction of PyMT with PP2A, Shc, or PI3K was affected by *Net1* deletion, we immunoprecipitated PyMT from wild-type and *Net1* knockout tumors and tested for coprecipitation of each protein. We observed that coprecipitation of the A and C subunits of PP2A was significantly impaired by *Net1* deletion, as was the coprecipitation of total and active Src, Shc, and p85 with PyMT (Fig. 5d). These data indicate that reduced activation of the Shc–ERK1/2 and PI3K–Akt pathways in *Net1* knockout tumors results from impaired recruitment of key signaling molecules to PyMT.

### *Net1* deletion inhibits tumor angiogenesis and lung metastasis in a tumor cell autonomous manner

**Fig. 6:**
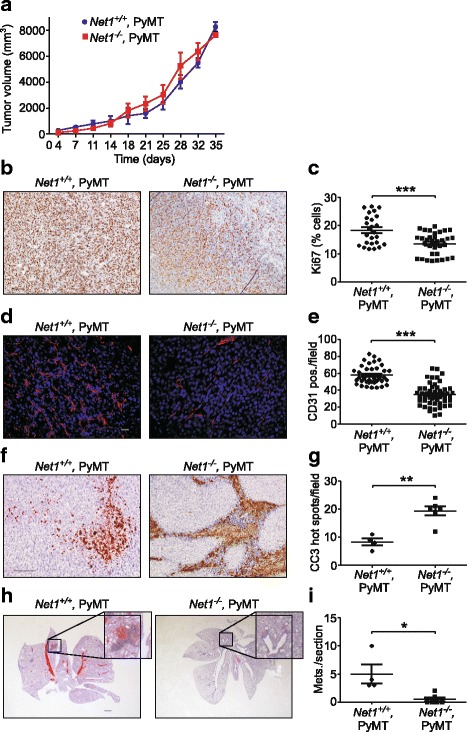
*Net1* deletion reduces proliferation and angiogenesis, and increases apoptosis in a tumor cell autonomous manner. Wild-type and *Net1* knockout PyMT tumor cells injected into fourth inguinal mammary gland of wild-type FVB mice. **a** Tumor growth. *Net1*^*+/+*^ = 4 mice; *Net1*^*−/−*^ = 6 mice. **b** Representative examples of Ki67 staining in *Net1*^*+/+*^ and *Net1*^*−/−*^ tumors. Bar = 100 μm. **c** Quantification of Ki67 staining in tumors. Five independent regions within each sample quantified from 4 *Net1*^*+/+*^ and 6 *Net1*^*−/−*^ tumors. Bars represent median values. **d** Representative examples of CD31 staining (red) in *Net1*^*+/+*^ and *Net1*^*−/−*^ tumors. DNA shown in blue. Bar = 20 μm. **e** Quantification of CD31 staining. Five independent regions from each tumor analyzed in 4 *Net1*^*+/+*^ and 6 *Net1*^*−/−*^ tumors. Bars represent median values. **f** Representative examples of CC3 staining in *Net1*^*+/+*^ and *Net1*^*−/−*^ tumors. Bar = 100 μm. **g** Quantification of CC3 staining. CC3 hot spots analyzed in 4 *Net1*^*+/+*^ and 6 *Net1*^*−/−*^ tumors. Bars represent median values. **h** Representative examples of lung metastasis in mice injected with *Net1*^*+/+*^ and *Net1*^*−/−*^ cells. Bar = 1000 μm. **i** Quantification of lung metastasis in 6 *Net1*^*+/+*^ and 6 *Net1*^*−/−*^ mice. Bars represent median values. *P < 0.05; **P < 0.01; ***P < 0.001. Mets metastases, Net1 neuroepithelial transforming gene 1, PyMT polyoma middle T antigen

To test whether the effects of *Net1* deletion on tumor angiogenesis and metastasis were tumor cell autonomous, primary MMTV-PyMT tumor cells were isolated from wild-type and *Net1* knockout mice and then transplanted into the mammary glands of syngeneic, wild-type FVB mice. Tumors were allowed to grow to the same size, at which time the mice were euthanized. Tumors and lungs were then excised to assess tumor characteristics and lung metastasis. We observed that PyMT tumors lacking *Net1* grew in volume at a similar rate to wild-type tumors (Fig. 6a). However, staining of sectioned tumors for Ki67 indicated that there was a small but significant decrease in the number of cells proliferating in *Net1* knockout tumors (Fig. 6b, c). When tumors were stained for CD31, we found that there were significantly fewer blood vessels in tumors lacking *Net1*, similar to what was observed in *Net1* knockout mice (Fig. 6d, e). Consistent with this observation, *Net1* knockout tumors generally were less well perfused and lacked blood vessels in the surrounding skin relative to wild-type tumors (Additional file 1: Figure S3). Staining for cleaved caspase 3 indicated that apoptosis was significantly increased in *Net1* knockout tumors (Fig. 6f, g). Moreover, there was a trend toward increased necrotic area in tumors derived from *Net1* knockout cells (Additional file 2: Figure S4). When we analyzed the lungs in mice with wild-type PyMT tumors we observed a significant degree of metastasis. This was generally less than that observed in the MMTV-PyMT genetic mouse model (Fig. 1d–h), but tumors in the cell injection model were only allowed to grow for 5 weeks. Importantly, lung metastasis was nearly absent in mice injected with PyMT cells lacking *Net1* (Fig. 6h, i). Taken together these data indicate that *Net1* deletion inhibits tumor angiogenesis and lung metastasis in a tumor cell autonomous nature.

### A Net1 gene expression signature predicts PI3K activation, cancer cell proliferation, and distant metastasis-free survival in human breast cancer patients

**Fig. 7:**
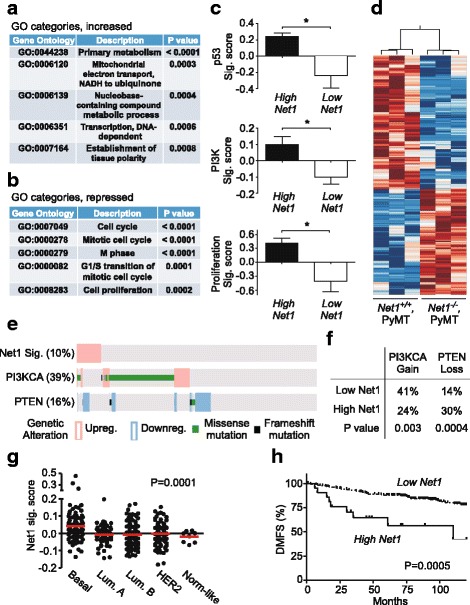
Identification of Net1-dependent gene signature in mammary gland tumors and analysis of human breast cancer patients. **a** Top 5 GO categories of genes activated in *Net1* knockout, PyMT tumors. **b** Top 5 GO categories of genes repressed in *Net1* knockout, PyMT tumors. **c** Signature scores in p53, PI3K, and proliferation gene expression pathways in wild-type (High Net1) or Net1 knockout (Low Net1) PyMT tumors. **d** Heat map of gene expression comprising Net1 signature in *Net1*^*+/+*^*,PyMT* and *Net1*^*−/−*^*,PyMT* tumors. **e** Frequency of Net1 gene expression signature, PI3KCA mutation or amplification, and PTEN loss of expression in human breast cancers in TCGA. **f** Correlation of Net1 signature with phosphatidylinositol 3-kinase catalytic subunit A (PIK3CA) overexpression or mutation, or PTEN loss, in human breast cancers. **g** Correlation of Net1 signature with human breast cancer subtypes. Statistical significance assessed by ANOVA. **h** Kaplan–Meier analysis of distant metastasis-free survival (DMFS) in human breast cancer patients with positive and negative Net1 gene expression signatures. GSE11121 analyzed. GO Gene Ontology, Lum. luminal, Net1 neuroepithelial transforming gene 1, PI3K phosphatidylinositol 3-kinase, PTEN phosphatase and tensin homolog, PyMT polyoma middle T antigen

To perform an unbiased analysis of the mechanism by which Net1 controls PyMT-stimulated tumorigenesis and metastasis, we analyzed global gene expression in wild-type and *Net1*^*−/−*^ PyMT mammary tumors. RNA was isolated from three tumors of each genotype and analyzed using an Illumina Bead Array for whole genome expression. Based on their expression patterns, we found that 241 genes were upregulated in the *Net1* knockout tumors while 166 genes were downregulated with a 1.5-fold change (*P* < 0.05) (Additional file 3: Table S1). Gene ontology analysis with GATHER [45] indicated that a significant portion of the genes activated in tumors from *Net1*^*−/−*^ mice corresponded to regulators of metabolism, mitochondrial function, transcription, and tissue polarity (Fig. 7a). This may reflect adaptation to altered energy demands caused by reduced tumor angiogenesis (Fig. 3f, g). On the other hand, the repressed genes corresponded to cell cycle and mitotic regulators (Fig. 7b), reflecting the reduced proliferation of *Net1*^*−/−*^ tumor cells (Fig. 2c, d).

To identify cancer pathways that are lost in the *Net1*^*−/−*^tumors, we predicted the activity of 52 pathways using previously published gene expression signatures [46] and approaches that we developed previously [32, 33]. A gene expression signature is a characteristic pattern in the transcriptional profile of a tumor that is indicative of the activation of a signaling pathway [47, 48]. Because they reflect the downstream consequences of pathway activity, gene expression signatures provide measures on the functional status of a pathway. Based on these signatures, we observed that three of those pathways, namely p53, PI3K, and proliferation, are consistently decreased in the *Net1* knockout tumors (Fig. 7c). Thus, *Net1*-expressing tumors correlated with the transcriptional profile of p53 mutant tumors, while *Net1*-deleted tumors correlated with transcription in wild-type p53-expressing tumors. Similarly, a PI3K transcriptional signature was repressed by *Net1* deletion, as was the proliferation-dependent gene expression signature (Fig. 7c). Thus, our observations indicating *Net1* dependence for cell survival, PI3K activity, and proliferation in PyMT-expressing tumors matched the apparent *Net1* dependence for these gene expression pathways.

Because of the importance of PI3K signaling to human breast cancer, we then assessed whether Net1 activation was associated with PI3K activity. To do this, we created a signature for Net1 activity using an empirical Bayes approach to identify the 277 genes (283 probes) that together can predict Net1 activation (Fig. 7d; Additional file 4: Figure S1; Additional file 5: Table S2). We then scored Net1 activity across breast cancer tumors from the TCGA [49] and correlated it with overexpression or mutagenic activation of the PI3K p110α catalytic subunit (PI3KCA) or deletion of the PIP_3_ phosphatase PTEN. We observed that 10% of all human breast cancers exhibited a high Net1 gene expression signature (Fig. 7e). Similar to previous results, 39% of human breast cancers exhibited PI3KCA activation, while 16% exhibited PTEN loss [49–51]. Importantly, patients with a high Net1 gene expression signature exhibited fewer instances of PI3KCA activation and increased incidence of PTEN loss (Fig. 7f). Coupled with our observation that PI3K signaling is high in wild-type *PyMT* tumors, this suggests that tumors with high Net1 activity did not require PI3KCA overexpression or mutagenic activation, but did tend to cosegregate with PTEN loss, to drive PI3K signaling.

When we assessed whether Net1 is activated in particular subtypes of breast cancer, we observed an increased incidence of a positive Net1 gene expression signature in basal-type breast cancers (Fig. 7g). This is consistent with other studies indicating that basal-type breast cancers tend to have a greater frequency of PI3K pathway activation [49]. We then examined whether a high Net1 gene expression signature correlated with distant metastasis-free survival in human breast cancer patients. We observed that patients with a high Net1 gene expression signature experienced reduced distant metastasis-free survival in a breast cancer dataset [52] (Fig. 7h), consistent with the requirement for *Net1* expression for metastasis in MMTV-PyMT mice. This result was confirmed in an independent dataset [53] (Additional file 6: Figure S2). Taken together, these data indicate that a Net1 gene expression signature is observed in highly proliferative breast tumors that harbor elevated PI3K signaling and tend to metastasize sooner, consistent with an important role for Net1 in disease progression in human breast cancer patients.

## Discussion

Although RhoA signaling is critically important for breast cancer cell motility and invasiveness in vitro, few studies have assessed its role in metastasis in vivo. In the present study we demonstrate that the RhoA subfamily GEF *Net1* contributes to PyMT-driven tumorigenesis and is required for efficient lung metastasis. Moreover, we demonstrate that high Net1 signaling correlates with increased human breast cancer metastasis, indicating that our findings are relevant to human disease progression. To our knowledge, this is the first report of a RhoA GEF promoting breast tumorigenesis or metastasis.

MMTV-PyMT mice model luminal B-type breast tumors [54]. However, the wide incidence of the Net1 gene expression signature in human breast cancers suggests that Net1 function is not limited to this breast cancer subtype. For example, we observed that a high Net1 gene expression signature correlated closely with human basal-type breast cancers (Fig. 7g). Moreover, the reduced distant metastasis-free survival in patients with a high Net1 gene expression signature was observed in a cohort of patients that was not subdivided according to cancer subtype (Fig. 7f). These findings, coupled with our previous results indicating that coexpression of Net1 with the β4 integrin predicted reduced distant metastasis-free survival and reduced overall survival in ERα-positive breast cancer patients [55], indicate that Net1 may promote metastasis in a wide range of breast cancer subtypes. The idea that aberrant RhoA activation drives breast cancer metastasis fits with the findings of others indicating that reduced expression of the RhoA subfamily-specific GAP DLC-1 is predictive of metastatic spread to the bone in all breast cancer subtypes [18]. These findings are distinct from studies focusing on Rac1 activators, which appear to function in a more subtype-specific manner. For example, the Rac1 GEFs P-Rex1 and Vav2/3 contribute to metastasis in luminal subtype breast cancers, while the Rac1 GEF Dock1 controls metastasis in HER2-positive breast cancers [19–21]. The subtype independence of Net1 has important implications for therapeutic approaches, as it suggests that targeting Net1 would be a widely applicable therapeutic strategy for breast cancer. Our data indicating that *Net1* deletion switched tumors to a wild-type p53 gene expression signature (Fig. 6c) may also suggest that targeting Net1 would sensitize breast tumors with wild-type p53 to chemotherapies dependent on p53 function.

A potential caveat of our Net1 gene expression signature is that it may contain components that reflect PyMT signaling, which would also be expected to include PI3K activation. Unfortunately, it is not technically possible to isolate the activity of one pathway from the function of the rest of the signaling network. In our design, we compare the gene expression profile of Net1 within a PyMT background, and, in principle, the contribution of PyMT should not be detected. Nevertheless, the notion that Net1 function is associated with PI3K signaling and metastasis is logical given the reported roles of Rho GTPase signaling in controlling PI3K activation and extracellular matrix invasion in cell-based studies. Future work will be required to dissect the components of the Net1 gene expression signature that are conserved among different breast cancer models.

It is unclear why there was not a significant increase in the survival of mice with *Net1* deletion, given the observed delay in tumor initiation. On the surface this would suggest that *Net1*-deleted tumors proliferated more rapidly, yet this was clearly not the case as Ki67 staining was reduced in both early and late tumors (Fig. 2), and unbiased gene expression analysis demonstrated significantly reduced expression of proliferation associated genes (Fig. 6b, c). This apparent contradiction most likely reflects the short delay in tumorigenesis (only 20 days), and the fact that *Net1*-deleted tumors tended to be fluid filled and less firm, which may have increased their apparent volume when measuring tumor size with calipers. The observation that they had larger necrotic cores supports this idea (Fig. 3a–c).

The *Net1* mouse model we used is a whole-body deletion of the *Net1* gene, so some of the phenotypes we observed may be due to cancer cell extrinsic as well as intrinsic effects. That being said, our tumor cell transplant experiments indicate that many of the phenotypes we observed are tumor cell autonomous. For example, tumors arising from injection of *Net1* knockout cells exhibited less proliferation, less angiogenesis, and increased apoptosis (Fig. 6b–g). Significantly, there was also less metastasis to the lungs (Fig. 6h, i), indicating that the decrease in metastasis in the genetic *Net1*^*−/−*^*,PyMT* mice was likely not the result of a delay in tumorigenesis. The decrease in proliferation in *Net1* knockout cells is likely attributable to decreased signaling by PyMT (Fig. 5). However, it is less clear how Net1 influences tumor angiogenesis. Presumably, *Net1* deletion inhibits the secretion of one or more angiogenic factors by the tumor cells. Whether this occurs through altered transcription, translation, or secretion is an open question, as RhoA signaling has been shown to impact each of these steps. Future work will be directed at understanding the mechanism by which Net1 controls tumor angiogenesis.

The PyMT oncogene is considered a general model for activated receptor tyrosine kinase (RTK) signaling in oncogenic transformation [39]. Thus, the effects of Net1 on PyMT signaling to PI3K and ERK1/2 may have wider applicability. This idea is supported by our finding that our *Net1*^*−/−*^ tumors have reduced gene expression signatures for PI3K and proliferation signaling (Fig. 7c). Signaling by PyMT is initiated through interactions with PP2A and Src, and these interactions are greatly reduced in *Net1*^*−/−*^ tumors (Fig. 5d). The mechanism by which Net1 regulates recruitment of PP2A and Src to PyMT is at present unclear, as there is no precedence for regulation of these events by Rho GTPases. *Net1* knockout tumor cells tended to express slightly more PyMT than wild-type cells (Figs. 2 and 5), so reduced recruitment cannot be due to effects on PyMT expression. One possibility is that loss of Net1 inhibits delivery of signaling molecules to the plasma membrane. RhoB has been shown previously to regulate EGF-mediated delivery of Src to the plasma membrane [56]. Moreover, RhoB has been reported to interact with the catalytic subunit of PP2A and to control its ability to recruit the B55 regulatory subunit [57, 58]. Thus, it may be that Net1-dependent recruitment of PP2A_A,C_ to PyMT is also RhoB dependent. In the future it will be important to test whether Net1 is required for recruitment of Src or PI3K to activated RTKs.

## Conclusions

These data indicate that *Net1* is important for PyMT-stimulated tumorigenesis and metastasis, and may also contribute to human breast cancer metastasis. Net1 contributes to breast cancer progression through multiple mechanisms, which include promotion of cancer cell proliferation and motility, and tumor angiogenesis.

## Additional files

Additional file 1: **Figure S3.** Representative examples of tumors from FVB mice injected with *Net1*^*+/+*^*,PyMT* and *Net1*^*−/−*^*,PyMT* cells. (PDF 353 kb)

Additional file 2: **Figure S4:** (**A**) Representative examples of H&E-stained tumor sections from FVB mice injected with *Net1*^*+/+*^*,PyMT* and *Net1*^*−/−*^*,PyMT* cells. (**B**) Quantification of necrotic areas from four *Net1*^*+/+*^*,PyMT* tumors and six *Net1*^*−/−*^*,PyMT* tumors. (PDF 315 kb)

Additional file 3: **Table S1.** Genes differentially expressed in *Net1+/+,PyMT* and *Net1−/−,PyMT* tumors, *P* < 0.05. (PDF 114 kb)

Additional file 4: **Figure S1.** Control analysis of the Net1 signature. (**A**) Signature score for *Net1+/+,PyMT* and *Net1−/−,PyMT* tumors. (**B**) Principal component analysis of gene expression for the Net1 signature. (PDF 17 kb)

Additional file 5: **Table S2.** Genes comprising the Net1 gene expression signature. Threshold for significance was a 5-fold change between Net1 wild-type and knockout tumors, *P* < 0.05. (PDF 36 kb)

Additional file 6: **Figure S2.** Correlation of the Net1 gene expression signature with reduced DMSF in breast cancer patients. GSE20685 analyzed. (PDF 13 kb)

## Abbreviations

AKT1: v-Akt murine thymoma viral oncogene homolog 1
CC3: Cleaved caspase 3
CD31: Cell determinant 31
CDC42: Cell division cycle 42
DLC-1: Deleted in liver cancer 1
DOCK1: Dedicator of cytokinesis 1
ERα: Estrogen receptor alpha
ERK1: Extracellular signal regulated kinase 1
FAK: Focal adhesion kinase
GST: Glutathione-S-transferase
Ki67: Marker of proliferation Ki-67
MLC2: Myosin regulatory light chain 2
MMTV: Mouse mammary tumor virus
MYPT1: Myosin phosphatase targeting subunit 1
Net1: Neuroepithelial transforming gene 1
p53: Tumor protein p53
p85: Phosphatidylinositol 3-kinase regulatory subunit p85α
PI3K: Phosphatidylinositol 3-kinase
PIP_3_: Phosphatidylinositol-3,4,5-phosphate
PLCγ: Phospholipase C gamma
PP2A: Protein phosphatase 2A
P-Rex1: Phosphatidylinositol-3,4,5-trisphosphate dependent Rac exchange factor 1
PTEN: Phosphatase and tensin homolog
PyMT: Polyoma middle T antigen
Rac1: Ras-Related C3 botulinum toxin substrate 1
RBD: RhoA binding domain
RhoA: Ras homolog family member A
RhoB: Ras homolog family member B
RhoC: Ras homolog family member C
RhoGAP: Rho GTPase activating protein
RhoGEF: Rho guanine nucleotide exchange factor
Shc: Src homology domain 2 containing
Src: SRC proto-oncogene, non-receptor tyrosine kinase
TCGA: The Cancer Genome Atlas
TGFβ: Transforming growth factor beta
Vav2: Vav guanine nucleotide exchange factor 2
