# Supplementary figures and images for: Contributions of the RhoA guanine nucleotide exchange factor Net1 to polyoma middle T antigen-mediated mammary gland tumorigenesis and metastasis
Source: Breast Cancer Res. 2018 May 16;20:41. doi: 10.1186/s13058-018-0966-2 (PMC5956559; doi:10.1186/s13058-018-0966-2)

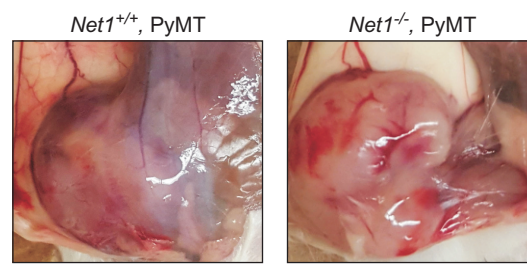

Supplement: Supplementary file 1 — Figure S3. Representative examples of tumors from FVB mice injected with Net1+/+,PyMT and Net1−/−,PyMT cells. (PDF 353 kb) [file 13058_2018_966_MOESM1_ESM.pdf]

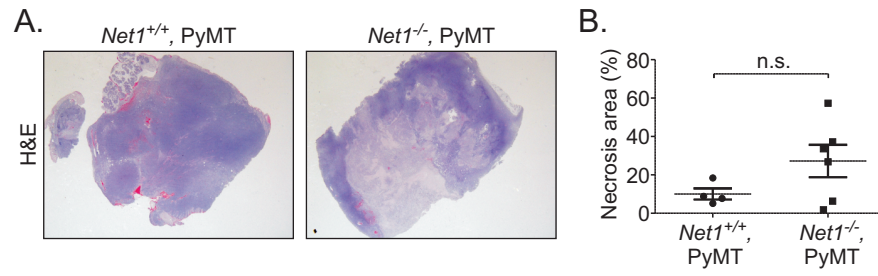

Supplement: Supplementary file 2 — Figure S4: (A) Representative examples of H&E-stained tumor sections from FVB mice injected with Net1+/+,PyMT and Net1−/−,PyMT cells. (B) Quantification of necrotic areas from four Net1+/+,PyMT tumors and six Net1−/−,PyMT tumors. (PDF 315 kb) [file 13058_2018_966_MOESM2_ESM.pdf]

A.

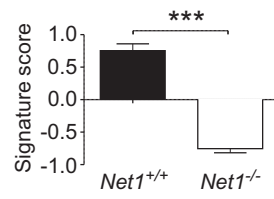

B.

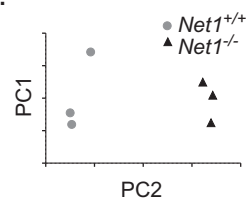

Supplement: Supplementary file 4 — Figure S1. Control analysis of the Net1 signature. (A) Signature score for Net1+/+,PyMT and Net1−/−,PyMT tumors. (B) Principal component analysis of gene expression for the Net1 signature. (PDF 17 kb) [file 13058_2018_966_MOESM4_ESM.pdf]

Zuo\_FigS2

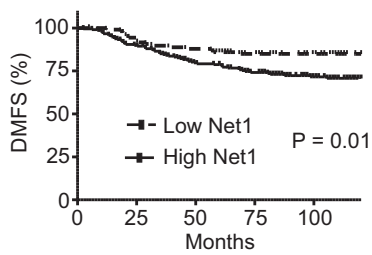

Supplement: Supplementary file 6 — Figure S2. Correlation of the Net1 gene expression signature with reduced DMSF in breast cancer patients. GSE20685 analyzed. (PDF 13 kb) [file 13058_2018_966_MOESM6_ESM.pdf]
