# Supplementary material for: Contributions of the RhoA guanine nucleotide exchange factor Net1 to polyoma middle T antigen-mediated mammary gland tumorigenesis and metastasis
Source: Breast Cancer Res. 2018 May 16;20:41. doi: 10.1186/s13058-018-0966-2 (PMC5956559; doi:10.1186/s13058-018-0966-2)
Supplement: Supplementary file 3 — Table S1. Genes differentially expressed in Net1+/+,PyMT and Net1−/−,PyMT tumors, P < 0.05. (PDF 114 kb) [file 13058_2018_966_MOESM3_ESM.pdf]

Table S1. Gene expression array results

All genes, P<0.05

TargetID	Signal_X	Signal_Y	Detection	Detection	Diff_Score	p-value	Fold_chan	Log_ratio(	Probe	SEARCH_K	ACCESSION	PROBE_ID	DEFINITION	SYNONYMS
CML3	22.9	0.4	0.0043	0.4177	-18.0628	0.015621	0.017467	-5.8392	6940753	ILMN_221	NM_0530	ILMN_273	Mus musc	4930583K21Rik; 4833414A14Rik
RAVER2	19.3	1.4	0.0085	0.3387	-17.4767	0.017878	0.072539	-3.7851	5360441	ILMN_214	NM_1830	ILMN_300	Mus musc	RP23-336G7.1; A430091O22Rik
9030224M	20.2	2.6	0.0064	0.2585	-16.5668	0.022046	0.128713	-2.95777	7650278	ILMN_209	NM_1777	ILMN_125	Mus musculus RIKEN cDNA 9030224M15 gene (9030224M15Rik),	
SLITRK4	58.3	7.9	0	0.0684	-20.1928	0.009566	0.135506	-2.88357	5550274	ILMN_219	NM_1787	ILMN_271	Mus musc	6430513G13; D930039I09Rik
DENND5B	20.7	2.9	0.0064	0.2489	-14.6792	0.034047	0.140097	-2.83551	2690348	ILMN_218	NM_1771	ILMN_270	Mus musc	9330160C06Rik
CIITA	25	3.9	0.0032	0.2051	-19.8307	0.010398	0.156	-2.68038	2630463	ILMN_217	NM_0075	ILMN_267	Mus musc	MGC130299; C2ta; CIITA
CDSN	31.8	5.1	0	0.1635	-16.5987	0.021884	0.160377	-2.64046	290333	ILMN_198	NM_0010	ILMN_125	Mus musc	AI747712
COMP	50.7	8.4	0	0.0598	-19.1511	0.012159	0.16568	-2.59352	3440538	ILMN_224	NM_0166	ILMN_2773395		
CPEB2	30.9	5.6	0	0.1239	-22.1154	0.006144	0.18123	-2.46411	2370707	ILMN_191	NM_1759	ILMN_258	Mus musc	A630055H10Rik
TNFRSF19	163.5	36.2	0	0	-13.4736	0.044941	0.221407	-2.17523	2060546	ILMN_188	NM_0138	ILMN_279	Mus musc	Tnfrsf20; TAJ; TAJ-ALPHA; AL023044; AW123854; Troy;
PRODH	222.7	49.6	0	0	-14.5844	0.034798	0.222721	-2.16669	3060400	ILMN_213	NM_0111	ILMN_2636666		
ATG9B	129.4	29.2	0	0.0011	-13.3351	0.046397	0.225657	-2.1478	3140682	ILMN_249	NM_0010	ILMN_316	Mus musc	Apg9l2; Nos3as; Apg912; sONE; eONE; Apgdc2; Gm574
LOC38616	53	12.8	0	0.0214	-13.1651	0.048249	0.241509	-2.04985	6400364	ILMN_200	XM_3591	ILMN_1236603		
N4BP2	48.4	12	0	0.0256	-27.6138	0.001732	0.247934	-2.01197	6770367	ILMN_261	NM_0010	ILMN_297	Mus musc	Gm868; E430014I16; N4bp2; Gm1791
SLC20A1	21.2	5.6	0.0053	0.1335	-13.1338	0.048598	0.264151	-1.92057	1340240	ILMN_206	AK084159	ILMN_1247410		
GM684	64.6	19.5	0	0.0075	-31.0779	0.00078	0.301858	-1.72806	2100022	ILMN_198	XM_9248	ILMN_121	PREDICTED: Mus musculus gene model 684, (NCBI) (Gm684), mR	
ZMYND10	42.9	13.1	0	0.0235	-17.2981	0.018629	0.305361	-1.71141	7510273	ILMN_185	NM_0532	ILMN_125	Mus musc	Blu
SLC5A5	59.5	18.3	0	0.0128	-13.0524	0.049518	0.307563	-1.70105	270086	ILMN_186	NM_0532	ILMN_1245214		
CKAP2L	41.5	13	0	0.0224	-19.0591	0.012419	0.313253	-1.6746	2450270	ILMN_214	NM_1815	ILMN_123	Mus musc	2610318C08Rik; AV070319; 2010016H04Rik
KCNA6	149.3	46.8	0	0	-24.2006	0.003801	0.313463	-1.67363	7000487	ILMN_209	NM_0135	ILMN_2596385		
IDI2	30.6	9.6	0	0.0491	-13.9427	0.040339	0.313725	-1.67243	2230356	ILMN_217	NM_1771	ILMN_121	Mus musc	IPPI2; 4833405L16Rik
GSG1L	97.9	30.8	0	0	-36.6985	0.000214	0.314607	-1.66838	5700164	ILMN_189	XM_91462	ILMN_246	PREDICTED: Mus musculus GSG1-like (Gsg1l), mRNA.	
LRP8	48.3	15.6	0	0.016	-23.4586	0.00451	0.322981	-1.63048	5960131	ILMN_204	NM_0010	ILMN_124	Mus musc	Lr8b; 4932703M08Rik; apoER2
ZFP276	48.5	16.3	0	0.0139	-23.4999	0.004467	0.336082	-1.57311	1770349	ILMN_187	NM_0204	ILMN_1222031		
CDC25A	52.3	17.8	0	0.0128	-20.463	0.008989	0.340344	-1.55493	3440100	ILMN_189	NM_0076	ILMN_277	Mus musc	D9Ertd393e
DNAJC14	27.2	9.3	0.0021	0.0491	-13.8976	0.040761	0.341912	-1.5483	2370594	ILMN_222	NM_0288	ILMN_275	Mus musc	DNAJ; LIP6; HDJ3; DRIP78; 5730551F12Rik
PITPNM3	25.7	8.8	0.0032	0.0598	-13.2337	0.047493	0.342412	-1.54619	1340167	ILMN_256	NM_0010	ILMN_311	Mus musc	AI848332; A330068P14Rik; Gm880
ATM	33.6	12.3	0	0.0235	-14.4862	0.035594	0.366071	-1.4498	4560551	ILMN_214	NM_0074	ILMN_278	Mus musc	C030026E19Rik; AI256621
PCDH21	1318.2	486	0	0	-43.8866	4.09E-05	0.368685	-1.43954	2060296	ILMN_216	NM_1308	ILMN_298	Mus musc	mKIAA1775; Prcad
EXTL1	202.1	76.1	0	0	-20.4388	0.009039	0.376546	-1.4091	1340133	ILMN_185	NM_0195	ILMN_1215877		
SLC27A3	143.9	55.9	0	0	-21.6015	0.006916	0.388464	-1.36415	6660747	ILMN_222	XM_1309	ILMN_2747857		
SLC38A3	46.2	18.2	0	0.0107	-17.2512	0.018831	0.393939	-1.34395	6100154	ILMN_222	NM_0238	ILMN_275	Mus musc	Slc38-3; 0610012J02Rik; Nat1; D9Ucla2
1700013G	55	22	0	0.0032	-15.7845	0.026397	0.4	-1.32193	4070497	ILMN_196	XM_8895	ILMN_122	PREDICTED: Mus musculus RIKEN cDNA 1700013G10 gene (17000	
EXTL1	297.3	120.9	0	0	-14.0799	0.039085	0.40666	-1.29811	7210669	ILMN_258	NM_0195	ILMN_287	Mus musc	D430033M16Rik
ADORA1	108.1	44.1	0	0	-22.3925	0.005764	0.407956	-1.29352	7510408	ILMN_216	NM_0010	ILMN_125	Mus musc	A1AR; A1R; BB176431; AI848715
C630028N	71.4	29.3	0	0.0011	-13.3655	0.046073	0.410364	-1.28502	2570100	ILMN_259	NM_1773	ILMN_296	Mus musculus RIKEN cDNA C630028N24 gene (C630028N24Rik),	
CML4	133.3	57	0	0	-19.3409	0.011639	0.427607	-1.22564	3170255	ILMN_223	NM_0234	ILMN_121	Mus musc	0610037O16Rik
TRP63	429.2	186	0	0	-18.0675	0.015605	0.433364	-1.20635	580064	ILMN_189	NM_0116	ILMN_121	Mus musc	p51/p63; Ket; TAp63; p63; MGC115972; AI462811; Trp
IL20RB	306.6	135.3	0	0	-16.5995	0.02188	0.441292	-1.1802	5050753	ILMN_246	NM_0010	ILMN_304	Mus musc	MGC130209; Fndc6; AV228068; Gm186
RFTN2	40.9	18.4	0	0.0118	-14.7577	0.033437	0.449878	-1.1524	6590243	ILMN_186	NM_0287	ILMN_121	Mus musc	3222401M22Rik; KIAA0084; 2700010E02Rik
MICALL2	98.3	44.6	0	0	-23.7376	0.004229	0.453713	-1.14015	6650349	ILMN_192	NM_1748	ILMN_271	Mus musc	JRAB; MICAL-L2; A930021H16Rik; FLJ00139; mFLJ0013
TCFAP2C	332	153.7	0	0	-15.4516	0.0285	0.462952	-1.11107	3780711	ILMN_223	NM_0093	ILMN_123	Mus musc	AA409384; Ap-2.2
ZFP295	46.1	21.6	0	0.0053	-15.1936	0.030244	0.468547	-1.09374	5420576	ILMN_192	NM_0010	ILMN_125	Mus musc	Znf295; B430213I24Rik; 5430437K12Rik; mKIAA1227
CRISPLD2	258.9	122.3	0	0	-19.5044	0.011209	0.472383	-1.08197	3780102	ILMN_208	NM_0302	ILMN_125	Mus musc	Lcrisp2; MGC116029; 1810049K24Rik
PIK3R3	157.7	74.5	0	0	-19.8981	0.010237	0.472416	-1.08187	1400184	ILMN_212	NM_1815	ILMN_124	Mus musc	p55pik; AA414954
D12ERTD5	132	62.4	0	0	-21.342	0.007342	0.472727	-1.08092	610491	ILMN_244	NM_0297	ILMN_298	Mus musc	2410157M17Rik; 9630047E15
KRT5	130.4	62.1	0	0	-15.2165	0.030085	0.476227	-1.07028	7150326	ILMN_221	NM_0270	ILMN_274	Mus musc	AW146334; Krt2-5; K5; 3300001P10Rik; Tfip8
POLD1	327.5	156.5	0	0	-17.3573	0.018377	0.477863	-1.06533	5220358	ILMN_215	NM_0111	ILMN_265	Mus musc	125kDa
USP37	62.2	30.1	0	0	-17.382	0.018273	0.483923	-1.04715	4210576	ILMN_184	NM_1769	ILMN_123	Mus musc	4932415L06Rik; C330008N13Rik
KCNA6	96.1	46.8	0	0	-16.4536	0.022628	0.486993	-1.03803	3140612	ILMN_209	NM_0135	ILMN_2760927		
LOC38061	84.4	41.3	0	0	-13.4513	0.045172	0.489336	-1.0311	2100561	ILMN_198	XM_14964	ILMN_1213986		
BACE1	469.7	230.2	0	0	-26.9024	0.002041	0.4901	-1.02885	580446	ILMN_215	NM_0117	ILMN_121	Mus musc	C76936
DYSF	68.7	34	0	0	-17.5131	0.017729	0.494905	-1.01478	7610398	ILMN_229	NM_0214	ILMN_313	Mus musc	mFLJ00175; 2310004N10Rik; D6Pas3; AI604795
PAPLN	928.9	461.9	0	0	-19.116	0.012257	0.497255	-1.00794	2680672	ILMN_219	NM_1308	ILMN_270	Mus musc	E030033C16Rik
ORF19	62.5	31.1	0	0	-13.9805	0.03999	0.4976	-1.00694	7150112	ILMN_210	NM_0268	ILMN_124	Mus musc	1110049F14Rik
ZFP334	50.6	25.2	0	0.0011	-14.6331	0.03441	0.498024	-1.00571	6060521	ILMN_184	NM_1784	ILMN_285	Mus musc	MGC27911; D2Ertd535e
TUBE1	54.3	27.2	0	0.0011	-15.1068	0.030855	0.500921	-0.99735	2600475	ILMN_252	NM_0280	ILMN_316	Mus musc	2310061K05Rik; Tube; AI551343
C330016O	46.6	23.4	0	0.0032	-13.6623	0.04303	0.502146	-0.99382	4070017	ILMN_217	NM_1459	ILMN_2685222		
BEX4	119.8	60.3	0	0	-17.0809	0.019584	0.503339	-0.9904	1410619	ILMN_231	NM_2124	ILMN_304	Mus musculus brain expressed gene 4 (Bex4), mRNA.	
ZFP296	69.6	35.1	0	0	-15.2653	0.029749	0.50431	-0.98762	5670154	ILMN_190	NM_0224	ILMN_291	Mus musc	2210018A16Rik
6430548M	505.5	258.9	0	0	-20.455	0.009005	0.512166	-0.96532	7160070	ILMN_188	NM_1722	ILMN_1219474		
SIDT1	715.9	366.8	0	0	-14.435	0.036016	0.512362	-0.96476	870156	ILMN_218	NM_1980	ILMN_124	Mus musc	B830021E24Rik; AW045928
SLC30A2	128.4	66.1	0	0	-15.3175	0.029393	0.514798	-0.95792	5130598	ILMN_220	XM_1317	ILMN_2718910		
LOC38640	316.2	163	0	0	-24.171	0.003827	0.515497	-0.95597	2230082	ILMN_200	XM_35921	ILMN_2538597		
CDC20	897.5	464.6	0	0	-15.2969	0.029533	0.51766	-0.94992	4610722	ILMN_211	NM_0232	ILMN_286	Mus musc	C87100; p55CDC; 2310042N09Rik
RPS6KL1	226.8	118.3	0	0	-22.013	0.006291	0.521605	-0.93897	3800327	ILMN_214	NM_1462	ILMN_264	Mus musc	A830084F09Rik; MGC38756; AW04644
ODZ4	106.5	55.6	0	0	-14.8717	0.032571	0.522066	-0.9377	3060382	ILMN_194	NM_0118	ILMN_316	Mus musc	Ten-m4; R75022; mKIAA1302; l7Rn3; l(7)-3Rn; Doc4; El
CDCA2	281.5	147.4	0	0	-16.1061	0.024513	0.523623	-0.9334	1820274	ILMN_212	NM_1753	ILMN_263	Mus musc	2610311M19Rik; AI586158
PCYOX1L	130.6	68.8	0	0	-16.9764	0.020061	0.526799	-0.92467	3870092	ILMN_217	NM_1728	ILMN_268	Mus musc	C630049M13; BC060677
MCM5	1017.1	537.1	0	0	-13.1106	0.048858	0.52807	-0.9212	6220270	ILMN_221	NM_0085	ILMN_274	Mus musc	AA617332; mCD46; AL033333; P1-CDC46; mCDC46; Cd
H2-AB1	357.4	191	0	0	-22.7156	0.005351	0.534415	-0.90397	5860435	ILMN_209	NM_2071	ILMN_291	Mus musc	Rmcs1; I-A<b>; H2-Ab; AI845868; I-Abeta; IAb; Ia-2; Ia2
PDZRN3	1255.7	673.6	0	0	-18.3533	0.014611	0.536434	-0.89853	6660201	ILMN_214	NM_0188	ILMN_315	Mus musc	1110020C07Rik; AL023082; LNX3; Semcap3; AI429718
CHI3L1	16924.2	9298.6	0	0	-20.6762	0.008558	0.549426	-0.864	150114	ILMN_210	NM_0076	ILMN_260	Mus musc	AW208766; brp-39; Brp39; Gp39
MCM10	255.2	140.3	0	0	-20.5563	0.008798	0.549765	-0.86311	4220326	ILMN_217	NM_0272	ILMN_297	Mus musc	C79164; AU018508; C330019M07Rik; 2410041F14Rik
BHLHB8	430.2	237.1	0	0	-14.1824	0.038173	0.551139	-0.85951	2750068	ILMN_214	NM_0108	ILMN_266	Mus musc	1810009C13Rik; Mist1
HS3ST3B1	210.7	116.8	0	0	-13.2927	0.046852	0.554343	-0.85115	2810603	ILMN_184	NM_0188	ILMN_125	Mus musc	Hs3st3b; 3Ost3b; 3-OST-3B; AW536289; 3OST3B1; m3-
3321401G	499.5	277.1	0	0	-14.959	0.031923	0.554755	-0.85008	5310156	ILMN_219	XM_1330	ILMN_1229200		
SPINT2	109.4	60.7	0	0	-16.8967	0.020433	0.554845	-0.84984	1170072	ILMN_214	NM_0114	ILMN_264	Mus musc	AL024025; HAI-2; C76321
E430003D	64.5	35.9	0	0	-13.5056	0.044611	0.556589	-0.84532	7570056	ILMN_203	AK088068	ILMN_1224932		
RCC1	130.4	73.5	0	0	-17.1705	0.019184	0.56365	-0.82713	5720626	ILMN_222	NM_1338	ILMN_274	Mus musc	Chc1; AI326872; 4931417M11Rik
DTX2	114.1	64.4	0	0	-16.4726	0.022529	0.564417	-0.82517	5890301	ILMN_217	NM_0237	ILMN_270	Mus musc	AU022494; Deltex2; AA408415; 2610524D08Rik
AACS	515.5	291	0	0	-14.4477	0.035911	0.5645	-0.82495	7650468	ILMN_216	NM_0302	ILMN_125	Mus musc	SUR5; 2210408B16Rik
IRS2	182.3	103.1	0	0	-18.1755	0.015221	0.565551	-0.82227	2100215	ILMN_231	NM_0010	ILMN_314	Mus musc	Irs-2
CCND1	941	532.8	0	0	-21.0089	0.007927	0.566206	-0.8206	20364	ILMN_210	NM_0076	ILMN_266	Mus musc	AI327039; PRAD1; bcl-1; Cyl-1

CDC7	149.9	85	0	0	-17.4738	0.01789	0.567045	-0.81847	110259	ILMN_218	NM_0098	ILMN_123	Mus musc	muCdc7; AI597260; Cdc7l1; MGC91067
APAF1	183.6	104.7	0	0	-17.9579	0.016003	0.570261	-0.8103	1990639	ILMN_248	NM_0010	ILMN_314	Mus musc	fog; 6230400I06Rik; Apaf-1; KIAA0413; Apaf1l; mKIAAC
HR	826.2	471.3	0	0	-15.2278	0.030007	0.570443	-0.80985	6330605	ILMN_218	NM_0218	ILMN_280	Mus musc	rh-bmh; AU; bldy; ba; N; rh; rhino; ALUNC
4632417K	193.1	110.2	0	0	-14.4283	0.036072	0.570689	-0.80922	1780279	ILMN_220	NM_0266	ILMN_271	Mus musc	AW413625
PEX6	790.9	451.7	0	0	-18.6499	0.013646	0.571122	-0.80813	2340537	ILMN_209	NM_1454	ILMN_271	Mus musc	mKIAA4177; D130055I09Rik; KIAA4177; AI132582; MG
RELL1	199.9	114.4	0	0	-17.4276	0.018082	0.572286	-0.80519	4560278	ILMN_189	NM_1459	ILMN_265	Mus musc	AA536743
RNPEP	949.5	546.3	0	0	-15.5881	0.027618	0.575355	-0.79747	7050307	ILMN_223	NM_1454	ILMN_1246024		
POF1B	224.2	129	0	0	-13.6074	0.043577	0.575379	-0.79742	6620368	ILMN_221	NM_1815	ILMN_124	Mus musc	2310066B14Rik
MTAP7D1	1080.2	622.4	0	0	-14.7891	0.033196	0.57619	-0.79538	670543	ILMN_219	NM_1449	ILMN_285	Mus musc	MGC28498; AV028413; MGC27585; Rprc1; BC019977;
GYLTL1B	1536.3	888.7	0	0	-15.8328	0.026105	0.578468	-0.78969	5900274	ILMN_216	NM_1726	ILMN_123	Mus musc	Large2; AI891893; Largel; mKIAA4105; KIAA4105; 5730
FAIM2	131.9	76.3	0	0	-16.179	0.024105	0.578469	-0.78969	1340746	ILMN_251	NM_0010	ILMN_313	Mus musc	mKIAA0950; NMP25; 2900002L20Rik; AI854036; lifegu
LIG1	357.6	207.3	0	0	-15.7864	0.026385	0.579698	-0.78663	3060767	ILMN_209	NM_0107	ILMN_259	Mus musc	LigI; AL033288
GALNTL4	83.1	48.4	0	0	-13.7154	0.042507	0.582431	-0.77984	7570360	ILMN_221	NM_1737	ILMN_273	Mus musc	2900011G21Rik; MGC32465; BC024988
MFSD7C	116.8	68.1	0	0	-14.1728	0.038258	0.583048	-0.77831	380132	ILMN_222	NM_1454	ILMN_124	Mus musc	MGC19050; CCT
MDN1	116.8	68.1	0	0	-13.639	0.043261	0.583048	-0.77831	3140326	ILMN_218	NM_0010	ILMN_126	Mus musc	A130070M06; 4833432B22Rik; AA958993; Gm135
KIF15	103.9	60.6	0	0	-14.8184	0.032973	0.583253	-0.77781	6330114	ILMN_212	NM_0106	ILMN_290	Mus musc	Knsl7; D330038N01; 3930402I10Rik; HKLP2; 3110023M
DYSF	430.1	252	0	0	-18.5685	0.013904	0.58591	-0.77125	1450068	ILMN_184	NM_0214	ILMN_242	Mus musc	mFLJ00175; 2310004N10Rik; D6Pas3; AI604795
PAOX	1975.7	1164.5	0	0	-13.165	0.04825	0.589411	-0.76265	3140102	ILMN_212	NM_1537	ILMN_279	Mus musc	AI118225; 2410012F02Rik; Pao
RNPEPL1	3037.9	1792.4	0	0	-18.2397	0.014998	0.590013	-0.76118	6200189	ILMN_215	NM_1814	ILMN_266	Mus musc	AI894167; 1110014H17Rik
RASSF10	130.3	77.3	0	0	-13.6404	0.043247	0.593246	-0.7533	5090703	ILMN_213	NM_1752	ILMN_263	Mus musculus Ras association (RalGDS/AF-6) domain family (N-te	
DDO	106.9	63.5	0	0	-14.2634	0.037468	0.594013	-0.75143	5960739	ILMN_190	NM_0274	ILMN_123	Mus musc	5330420D20Rik; 5730402C02Rik; AI467244
MPRIP	264.4	157.3	0	0	-17.0548	0.019702	0.594932	-0.7492	1300674	ILMN_186	NM_2012	ILMN_125	Mus musc	9530046C02; RP23-180B18.4; MGC67316; p116Rip; AI
ALDH5A1	580.7	345.6	0	0	-18.1526	0.015302	0.595144	-0.74869	2070243	ILMN_188	NM_1725	ILMN_266	Mus musc	Ssdh1; 6330403E24Rik; D630032B01Rik; Ahd-1; Ahd1
5730402C	141.6	84.4	0	0	-15.2669	0.029738	0.596045	-0.74651	6590070	ILMN_190	NM_0274	ILMN_2472708		
LOC54738	279.6	166.8	0	0	-14.5362	0.035187	0.596567	-0.74525	1690368	ILMN_223	XR_03089	ILMN_275	PREDICTED: Mus musculus similar to castor homolog 1, zinc finger	
CDKN2C	258.8	154.4	0	0	-16.871	0.020554	0.5966	-0.74516	7150528	ILMN_213	NM_0076	ILMN_122	Mus musc	INK4d; C77269; INK4c; p18INK4c; p18
BCKDK	1111	663.6	0	0	-14.7733	0.033317	0.5973	-0.74347	3890113	ILMN_214	NM_0097	ILMN_124	Mus musc	AI327402
ARFGEF2	101.6	60.7	0	0	-13.7945	0.04174	0.597441	-0.74313	1690639	ILMN_190	XM_13064	ILMN_1230252		
NUP210	732.9	439.1	0	0	-17.5351	0.01764	0.599127	-0.73907	1230703	ILMN_211	NM_0188	ILMN_125	Mus musc	Pom210; 9830001L10; gp210; AI836801; gp190
ACVR2B	259.1	155.4	0	0	-16.6561	0.021597	0.599768	-0.73752	2140706	ILMN_223	NM_0073	ILMN_123	Mus musc	MGC118477; ActRIIB
SLC5A6	246.2	148	0	0	-16.4531	0.02263	0.601137	-0.73423	6200762	ILMN_220	NM_1778	ILMN_122	Mus musc	E430023I20; MGC109689
4931426K	116.4	70.1	0	0	-14.1668	0.038311	0.602234	-0.7316	2100414	ILMN_196	XM_35726	ILMN_2701129		
TLE6	654.8	395.2	0	0	-17.5935	0.017404	0.603543	-0.72847	780445	ILMN_217	NM_0532	ILMN_290	Mus musc	Grg6; 1810057E06Rik
GPC1	962.7	587.6	0	0	-13.2063	0.047794	0.610367	-0.71225	7210039	ILMN_184	NM_0166	ILMN_263	Mus musc	AI462976
CDAN1	147.2	89.9	0	0	-14.4032	0.036281	0.610734	-0.71138	2680450	ILMN_247	NM_0268	ILMN_295	Mus musc	CDA1; MGC144128; AI448026; 1500015A01Rik; CDA-I;
SH3KBP1	826.9	506.9	0	0	-14.0585	0.039278	0.613012	-0.70601	520762	ILMN_219	NM_0213	ILMN_275	Mus musc	Ruk; AI447724; Seta; 1200007H22Rik; 5830464D22Rik;
HSPG2	340.4	208.9	0	0	-16.0855	0.024629	0.61369	-0.70442	7400520	ILMN_217	XM_97887	ILMN_268	PREDICTED: Mus musculus perlecan (heparan sulfate proteoglyca	
PPP4C	2194.4	1346.7	0	0	-13.0031	0.050083	0.613699	-0.7044	6270735	ILMN_214	NM_0196	ILMN_123	Mus musc	1110002D08Rik; Ppx; AU016079
PIK3R3	576.9	354.3	0	0	-14.5819	0.034818	0.614145	-0.70335	1850446	ILMN_212	NM_1815	ILMN_122	Mus musc	p55pik; AA414954
LOC10004	831.2	510.8	0	0	-16.495	0.022413	0.614533	-0.70244	3180379	ILMN_213	XM_00148	ILMN_125	PREDICTED: Mus musculus hypothetical protein LOC100043671 (L	
TJP3	110.3	67.8	0	0	-13.1797	0.048087	0.614687	-0.70208	520754	ILMN_220	NM_0137	ILMN_2723523		
LIG3	1119.1	688.7	0	0	-16.9721	0.020081	0.615405	-0.70039	5860561	ILMN_186	NM_0107	ILMN_1256377		
KCNN4	1690.4	1041	0	0	-14.9583	0.031928	0.615831	-0.69939	4150224	ILMN_223	NM_0084	ILMN_276	Mus musc	IK1; SK4; KCA4; IKCA1; mIKCa1
KIF2C	192.2	118.4	0	0	-14.8322	0.032869	0.616025	-0.69894	1780543	ILMN_217	NM_1344	ILMN_121	Mus musc	MCAK; X83316; MGC11883; 4930402F02Rik; Knsl6; EST
NOTCH1	319.5	197	0	0	-13.6214	0.043437	0.616588	-0.69762	6450022	ILMN_218	NM_0087	ILMN_124	Mus musc	Tan1; Mis6; 9930111A19Rik; lin-12
IGSF9	201.5	124.5	0	0	-13.2989	0.046785	0.617866	-0.69463	6400184	ILMN_250	NM_0336	ILMN_280	Mus musc	Dasm1; Ncaml; mKIAA1355; Kiaa1355-hp; NRT1; 644E
SUV420H2	506.2	313	0	0	-16.2161	0.0239	0.618333	-0.69354	3120367	ILMN_215	NM_1461	ILMN_126	Mus musc	MGC36471; BC024816; Suv4-20h2
ARHGEF6	1906.9	1181	0	0	-16.8444	0.02068	0.61933	-0.69122	5260035	ILMN_213	NM_1528	ILMN_263	Mus musc	4930592P22Rik; 1700038J06Rik; 1600028C08Rik
GM1673	185	114.9	0	0	-14.3897	0.036394	0.621081	-0.68715	4830519	ILMN_200	NM_0010	ILMN_123	Mus musculus gene model 1673, (NCBI) (Gm1673), mRNA. XM_92	
CYBASC3	204.9	128.1	0	0	-14.365	0.036602	0.625183	-0.67765	6370739	ILMN_208	NM_2013	ILMN_259	Mus musc	BC065078; MGC86114
2010317E	278.1	173.9	0	0	-14.925	0.032174	0.625315	-0.67735	3180438	ILMN_214	XM_13005	ILMN_1218312		
1600021P	136.6	85.5	0	0	-13.0348	0.049719	0.625915	-0.67596	6280386	ILMN_188	NM_1777	ILMN_245	Mus musc	A430031N04; C87006; MGC103296
CDC20	508.8	319.3	0	0	-15.54	0.027925	0.627555	-0.67219	4390228	ILMN_211	NM_0232	ILMN_261	Mus musc	C87100; p55CDC; 2310042N09Rik
UHRF1	1193.8	749.4	0	0	-16.045	0.02486	0.627743	-0.67175	4560397	ILMN_193	NM_0109	ILMN_251	Mus musc	AL022808; Np95; ICBP90; RNF106
ZFP259	914.2	574.5	0	0	-15.8784	0.025832	0.628418	-0.6702	3290091	ILMN_185	NM_0117	ILMN_124	Mus musc	AI303781; ZPR1
2310004N	1414	889.4	0	0	-16.0047	0.025092	0.628996	-0.66888	1980348	ILMN_222	NM_0288	ILMN_2777175		
LOC10004	236.3	148.7	0	0	-14.3789	0.036485	0.629285	-0.66821	450300	ILMN_190	XM_00147	ILMN_271	PREDICTED: Mus musculus similar to Nuclear receptor coactivator	
LOC10004	3182.9	2006.8	0	0	-16.0708	0.024713	0.630494	-0.66544	6940521	ILMN_215	XM_00147	ILMN_265	PREDICTED: Mus musculus similar to Hmgcs1 protein, transcript v	
UBA1	1742.5	1099	0	0	-14.6608	0.034192	0.630703	-0.66497	6250022	ILMN_185	NM_0094	ILMN_243	Mus musc	A1S9; AA989744; Ube-1; Sbx
PRLR	866.8	550	0	0	-15.389	0.028913	0.634518	-0.65627	5560722	ILMN_210	NM_0111	ILMN_261	Mus musc	Pr-3; Prlr-rs1; AI987712; Pr-1
FCHSD2	618.6	394.6	0	0	-14.9443	0.032031	0.637892	-0.64862	5260079	ILMN_219	NM_1990	ILMN_279	Mus musc	BC034086; Sh3md3; R74866; mKIAA0769
ATAD3A	1136.7	725.1	0	0	-13.9849	0.039949	0.637899	-0.6486	6270059	ILMN_218	NM_1792	ILMN_282	Mus musc	KIAA1273; Tob3; 2400004H09Rik; mKIAA1273
GTSE1	255.1	163.2	0	0	-13.8116	0.041576	0.639749	-0.64442	1500148	ILMN_215	NM_0138	ILMN_290	Mus musc	Gtse-1; B99
2310022A	302.5	193.6	0	0	-14.0631	0.039236	0.64	-0.64386	7570612	ILMN_224	NM_1751	ILMN_1227904		
MUM1	801.2	513.8	0	0	-14.854	0.032704	0.641288	-0.64096	6110242	ILMN_209	NM_0234	ILMN_121	Mus musc	9430059D04Rik; 2610019J09; UBE-1C2
IGF2R	417.3	268	0	0	-14.2935	0.037209	0.642224	-0.63885	670754	ILMN_208	NM_0105	ILMN_293	Mus musc	Mpr300; CD222; AI661837; CI-MPR; M6P/IGF2R
SYVN1	2384.5	1533.3	0	0	-15.0789	0.031053	0.643028	-0.63705	10674	ILMN_212	NM_0287	ILMN_262	Mus musc	D530017H19Rik; Hrd1; AW211966; 1200010C09Rik; C8
TMEM51	1509.1	971.5	0	0	-14.9254	0.032171	0.643761	-0.6354	2750594	ILMN_208	NM_1454	ILMN_258	Mus musc	BC003277; FLJ10199; RP23-103K12.2
HOMER1	222.1	143.3	0	0	-13.2296	0.047538	0.645205	-0.63217	3870040	ILMN_211	NM_1521	ILMN_122	Mus musc	SYN47; Ves-1; PSD-Zip45
GLG1	1943	1255.3	0	0	-14.8192	0.032967	0.646063	-0.63025	7320136	ILMN_186	NM_0091	ILMN_125	Mus musc	CFR-1; CFR; MG160; AI593353; Selel; MG-160; AW537
4931433E	515	333.5	0	0	-13.6833	0.042822	0.647573	-0.62689	520487	ILMN_188	XM_48476	ILMN_2451795		
BUB1B	244.5	159.1	0	0	-13.0513	0.04953	0.650716	-0.6199	2350095	ILMN_217	NM_0097	ILMN_2774007		
HOMER1	357.5	233.3	0	0	-13.422	0.045478	0.652587	-0.61576	2260722	ILMN_211	NM_1521	ILMN_271	Mus musc	SYN47; Ves-1; PSD-Zip45
NCAPH	282.9	184.7	0	0	-13.1267	0.048678	0.652881	-0.61511	6200367	ILMN_229	NM_1448	ILMN_279	Mus musc	MGC38424; mKIAA0074; HCAP-H; A730011O11Rik; Brr
HIST1H2AI	5377.8	3520.8	0	0	-14.3256	0.036935	0.654692	-0.61111	4250711	ILMN_196	NM_1756	ILMN_121	Mus musc	H2a-221
SKP2	366.4	240	0	0	-13.2997	0.046777	0.655022	-0.61039	1710377	ILMN_185	NM_1454	ILMN_125	Mus musc	FBXL1; MGC102075; MGC116668
CDC20	325.8	213.8	0	0	-13.0959	0.049024	0.656231	-0.60772	4570088	ILMN_211	NM_0232	ILMN_286	Mus musc	C87100; p55CDC; 2310042N09Rik
EIF2B4	1351.1	887.4	0	0	-13.9585	0.040193	0.656798	-0.60648	5570376	ILMN_220	NM_0101	ILMN_272	Mus musc	Eif2b
CENPA	2655.1	1744.1	0	0	-13.8234	0.041463	0.656887	-0.60628	7200519	ILMN_223	NM_0076	ILMN_123	Mus musc	Cenp-A
MYO9B	491.4	323.2	0	0	-13.3925	0.045788	0.657713	-0.60447	4570309	ILMN_211	NM_0157	ILMN_261	Mus musculus myosin IXb (Myo9b), mRNA.	
B930041F	657.2	434.6	0	0	-13.3473	0.046267	0.66129	-0.59664	4900220	ILMN_212	NM_1786	ILMN_125	Mus musc	MNCb-3966
PIGH	425.4	281.5	0	0	-13.0012	0.050105	0.66173	-0.59569	2370563	ILMN_211	NM_0299	ILMN_123	Mus musc	A930028P05Rik; MGC102258; 2210416H01Rik
NENF	908.9	1322	0	0	13.2025	0.047835	1.454505	0.540529	2230403	ILMN_218	NM_0254	ILMN_294	Mus musc	SCIRP10; 1110060M21Rik; Spuf
C1QC	632.7	925.1	0	0	13.3428	0.046315	1.462146	0.548088	3710170	ILMN_219	NM_0075	ILMN_271	Mus musc	Ciqc; C1qg; AI385742
CPNE8	301.5	442.3	0	0	13.0179	0.049913	1.466998	0.552867	6290528	ILMN_232	NM_0010	ILMN_315	Mus musc	1200003E11Rik; 1500031E20Rik
VPS25	796.8	1176	0	0	13.9368	0.040394	1.475904	0.561599	4860181	ILMN_219	NM_0267	ILMN_122	Mus musc	D11Wsu68e; AW107467; 1110020N13Rik
CYHR1	412.4	608.9	0	0	13.6133	0.043518	1.476479	0.562161	3390605	ILMN_219	NM_1809	ILMN_122	Mus musc	AU042374; 1110031M01Rik; Chrp
