# Supplementary material for: Contributions of the RhoA guanine nucleotide exchange factor Net1 to polyoma middle T antigen-mediated mammary gland tumorigenesis and metastasis
Source: Breast Cancer Res. 2018 May 16;20:41. doi: 10.1186/s13058-018-0966-2 (PMC5956559; doi:10.1186/s13058-018-0966-2)
Supplement: Supplementary file 5 — Table S2. Genes comprising the Net1 gene expression signature. Threshold for significance was a 5-fold change between Net1 wild-type and knockout tumors, P < 0.05. (PDF 36 kb) [file 13058_2018_966_MOESM5_ESM.pdf]

**Table S2. Genes differentially expressed for Bayes analysis**

| Gene ID | Gene Nam | p.value | FDR | Num Sam | Num Sam | Mean NET | Mean WT | Log_2 Fol | Direction | NET1_KO | NET1_KO | NET1_KO | WT_1 | WT_2 | WT_3 |
|---|---|---|---|---|---|---|---|---|---|---|---|---|---|---|---|
| ILMN_121 | Gypc | 9.51E-06 | 0.134233 | 3 | 3 | 3.693126 | 0 | 3.693126 | Higher in N | 3.523562 | 3.877744 | 3.678072 | 0 | 0 | 0 |
| ILMN_260 | Haao | 2.32E-05 | 0.134233 | 3 | 3 | 0 | 3.056567 | 3.056567 | Higher in W | 0 | 0 | 0 | 2.981853 | 3.017922 | 3.169925 |
| ILMN_258 | Col6a1 | 2.85E-05 | 0.134233 | 3 | 3 | 3.084324 | 0 | 3.084324 | Higher in N | 3.277985 | 3.087463 | 2.887525 | 0 | 0 | 0 |
| ILMN_263 | Ubqln3 | 3.83E-05 | 0.134233 | 3 | 3 | 3.418223 | 0 | 3.418223 | Higher in N | 3.336283 | 3.847997 | 3.070389 | 0 | 0 | 0 |
| ILMN_311 | Henmt1 | 4.56E-05 | 0.134233 | 3 | 3 | 2.864631 | 0 | 2.864631 | Higher in N | 3.035624 | 2.632268 | 2.925999 | 0 | 0 | 0 |
| ILMN_125 | Fam63a | 4.68E-05 | 0.134233 | 3 | 3 | 2.857635 | 0 | 2.857635 | Higher in N | 2.632268 | 3.053111 | 2.887525 | 0 | 0 | 0 |
| ILMN_122 | Rab3gap2 | 4.76E-05 | 0.134233 | 3 | 3 | 3.173474 | 0.087678 | 3.085796 | Higher in N | 2.847997 | 3.350497 | 3.321928 | 0.263034 | 0 | 0 |
| ILMN_316 | Defb48 | 5.55E-05 | 0.134233 | 3 | 3 | 2.729035 | 0 | 2.729035 | Higher in N | 2.536053 | 2.744161 | 2.906891 | 0 | 0 | 0 |
| ILMN_122 | Ccnd2 | 5.76E-05 | 0.134233 | 3 | 3 | 0 | 2.576193 | 2.576193 | Higher in W | 0 | 0 | 0 | 2.510962 | 2.608809 | 2.608809 |
| ILMN_314 | Armc3 | 5.77E-05 | 0.134233 | 3 | 3 | 3.29025 | 0 | 3.29025 | Higher in N | 3.419539 | 3.643856 | 2.807355 | 0 | 0 | 0 |
| ILMN_261 | Asph | 6.02E-05 | 0.134233 | 3 | 3 | 0.087678 | 3.283877 | 3.196199 | Higher in W | 0 | 0 | 0.263034 | 3.277985 | 2.906891 | 3.666757 |
| ILMN_262 | Aicda | 6.08E-05 | 0.134233 | 3 | 3 | 2.656476 | 0 | 2.656476 | Higher in N | 2.560715 | 2.560715 | 2.847997 | 0 | 0 | 0 |
| ILMN_123 | Smg5 | 6.42E-05 | 0.134233 | 3 | 3 | 0.161809 | 3.518992 | 3.357183 | Higher in W | 0 | 0 | 0.485427 | 3.121015 | 3.887525 | 3.548437 |
| ILMN_304 | Zdhhc19 | 7.57E-05 | 0.134233 | 3 | 3 | 0 | 2.614494 | 2.614494 | Higher in W | 0 | 0 | 0 | 2.847997 | 2.459432 | 2.536053 |
| ILMN_122 | Jarid2 | 8.2E-05 | 0.134233 | 3 | 3 | 0 | 3.228348 | 3.228348 | Higher in W | 0 | 0 | 0 | 2.678072 | 3.446256 | 3.560715 |
| ILMN_124 | C330021F | 8.36E-05 | 0.134233 | 3 | 3 | 0 | 3.789269 | 3.789269 | Higher in W | 0 | 0 | 0 | 4.314697 | 4 | 3.053111 |
| ILMN_125 | Zfhx4 | 8.49E-05 | 0.134233 | 3 | 3 | 0 | 2.676308 | 2.676308 | Higher in W | 0 | 0 | 0 | 2.944858 | 2.678072 | 2.405992 |
| ILMN_246 | Kdm4a | 8.93E-05 | 0.134233 | 3 | 3 | 0 | 2.61725 | 2.61725 | Higher in W | 0 | 0 | 0 | 2.906891 | 2.485427 | 2.459432 |
| ILMN_266 | Armc3 | 9.43E-05 | 0.134233 | 3 | 3 | 3.55924 | 0.087678 | 3.471562 | Higher in N | 4.129283 | 3 | 3.548437 | 0 | 0 | 0.263034 |
| ILMN_124 | Cpox | 9.56E-05 | 0.134233 | 3 | 3 | 2.995565 | 0 | 2.995565 | Higher in N | 3.321928 | 3.153805 | 2.510962 | 0 | 0 | 0 |
| ILMN_124 | Deaf1 | 9.94E-05 | 0.134233 | 3 | 3 | 0 | 2.592333 | 2.592333 | Higher in W | 0 | 0 | 0 | 2.510962 | 2.887525 | 2.378512 |
| ILMN_277 | Nodal | 0.000105 | 0.134233 | 3 | 3 | 2.856821 | 0 | 2.856821 | Higher in N | 2.678072 | 3.307429 | 2.584963 | 0 | 0 | 0 |
| ILMN_121 | Aif1 | 0.000105 | 0.134233 | 3 | 3 | 0 | 2.365508 | 2.365508 | Higher in W | 0 | 0 | 0 | 2.292782 | 2.510962 | 2.292782 |
| ILMN_259 | Nipsnap3b | 0.000105 | 0.134233 | 3 | 3 | 3.045322 | 0.045835 | 2.999487 | Higher in N | 3.321928 | 2.536053 | 3.277985 | 0 | 0.137504 | 0 |
| ILMN_281 | Scn4a | 0.000109 | 0.134233 | 3 | 3 | 0 | 2.382093 | 2.382093 | Higher in W | 0 | 0 | 0 | 2.560715 | 2.292782 | 2.292782 |
| ILMN_264 | Ier5 | 0.00011 | 0.134233 | 3 | 3 | 0 | 2.933133 | 2.933133 | Higher in W | 0 | 0 | 0 | 2.963474 | 2.485427 | 3.350497 |
| ILMN_265 | Akt2 | 0.000114 | 0.134233 | 3 | 3 | 0 | 2.975723 | 2.975723 | Higher in W | 0 | 0 | 0 | 3.446256 | 2.536053 | 2.944858 |
| ILMN_272 | Ercc4 | 0.000124 | 0.140274 | 3 | 3 | 0.282666 | 3.411029 | 3.128363 | Higher in W | 0 | 0.847997 | 0 | 3.247928 | 3.364572 | 3.620586 |
| ILMN_122 | Hrk | 0.000139 | 0.151899 | 3 | 3 | 3.151103 | 0.342856 | 2.808246 | Higher in N | 3.035624 | 3.364572 | 3.053111 | 0.263034 | 0.765535 | 0 |
| ILMN_287 | Gm906 | 0.000153 | 0.156671 | 3 | 3 | 0.087678 | 2.984242 | 2.896564 | Higher in W | 0.263034 | 0 | 0 | 3.307429 | 3.185867 | 2.459432 |
| ILMN_124 | Mpo | 0.00016 | 0.156671 | 3 | 3 | 0 | 2.346467 | 2.346467 | Higher in W | 0 | 0 | 0 | 2.070389 | 2.432959 | 2.536053 |
| ILMN_123 | Ptgs2 | 0.000163 | 0.156671 | 3 | 3 | 2.371705 | 0 | 2.371705 | Higher in N | 2.632268 | 2.378512 | 2.104337 | 0 | 0 | 0 |
| ILMN_125 | Zwilch | 0.000173 | 0.16117 | 3 | 3 | 0.194988 | 3.357333 | 3.162345 | Higher in W | 0.584963 | 0 | 0 | 3.584963 | 2.786596 | 3.70044 |
| ILMN_274 | Ctag2 | 0.000178 | 0.161243 | 3 | 3 | 2.991505 | 0.161809 | 2.829696 | Higher in N | 2.827819 | 2.70044 | 3.446256 | 0 | 0 | 0.485427 |
| ILMN_123 | Ampd1 | 0.000203 | 0.168945 | 3 | 3 | 2.918296 | 0.421011 | 2.497284 | Higher in N | 2.906891 | 2.847997 | 3 | 0.678072 | 0 | 0.584963 |
| ILMN_293 | Slc39a12 | 0.000217 | 0.168945 | 3 | 3 | 2.955776 | 0 | 2.955776 | Higher in N | 2.722466 | 3.608809 | 2.536053 | 0 | 0 | 0 |
| ILMN_269 | Srrm1 | 0.00022 | 0.168945 | 3 | 3 | 0 | 2.772402 | 2.772402 | Higher in W | 0 | 0 | 0 | 3.350497 | 2.405992 | 2.560715 |
| ILMN_123 | Slc30a7 | 0.000226 | 0.168945 | 3 | 3 | 2.64382 | 0 | 2.64382 | Higher in N | 2.201634 | 3.121015 | 2.608809 | 0 | 0 | 0 |
| ILMN_276 | Fam107a | 0.000235 | 0.168945 | 3 | 3 | 2.983664 | 0 | 2.983664 | Higher in N | 3.336283 | 2.292782 | 3.321928 | 0 | 0 | 0 |
| ILMN_123 | Gdf1 /// Ce | 0.000253 | 0.168945 | 3 | 3 | 0.58282 | 3.427812 | 2.844991 | Higher in W | 1.070389 | 0 | 0.678072 | 3.364572 | 3.321928 | 3.596935 |
| ILMN_289 | Garem | 0.00026 | 0.168945 | 3 | 3 | 3.26423 | 0.255178 | 3.009052 | Higher in N | 3.053111 | 3.776104 | 2.963474 | 0 | 0.765535 | 0 |
| ILMN_244 | Ccdc74a | 0.000261 | 0.168945 | 3 | 3 | 2.682164 | 0 | 2.682164 | Higher in N | 2.350497 | 2.432959 | 3.263034 | 0 | 0 | 0 |
| ILMN_125 | Sorbs1 | 0.000263 | 0.168945 | 3 | 3 | 3.019583 | 0.255178 | 2.764405 | Higher in N | 3 | 3.336283 | 2.722466 | 0 | 0 | 0.765535 |
| ILMN_285 | Gkn3 | 0.000267 | 0.168945 | 3 | 3 | 0 | 2.57048 | 2.57048 | Higher in W | 0 | 0 | 0 | 2.827819 | 2.847997 | 2.035624 |
| ILMN_247 | Zfp422 | 0.000279 | 0.169319 | 3 | 3 | 0.087678 | 2.488277 | 2.400599 | Higher in W | 0 | 0 | 0.263034 | 2.137504 | 2.867896 | 2.459432 |
| ILMN_262 | Rtn4r | 0.000307 | 0.17393 | 3 | 3 | 3.271577 | 0.450166 | 2.821411 | Higher in N | 3.104337 | 3.765535 | 2.944858 | 0 | 0.765535 | 0.584963 |
| ILMN_307 | Lrrc48 | 0.000324 | 0.180084 | 3 | 3 | 0.249487 | 3.390452 | 3.140965 | Higher in W | 0 | 0.485427 | 0.263034 | 3.797013 | 3.765535 | 2.608809 |
| ILMN_274 | Ptdss1 | 0.000336 | 0.183411 | 3 | 3 | 2.944385 | 0.194988 | 2.749398 | Higher in N | 3.364572 | 3.035624 | 2.432959 | 0 | 0.584963 | 0 |
| ILMN_121 | Siah1b | 0.000358 | 0.192264 | 3 | 3 | 2.589701 | 0 | 2.589701 | Higher in N | 3.153805 | 2.104337 | 2.510962 | 0 | 0 | 0 |
| ILMN_293 | Spryd7 | 0.000414 | 0.208285 | 3 | 3 | 2.557475 | 0 | 2.557475 | Higher in N | 2 | 3.087463 | 2.584963 | 0 | 0 | 0 |
| ILMN_242 | Gabrg3 | 0.000414 | 0.208285 | 3 | 3 | 0 | 2.781675 | 2.781675 | Higher in W | 0 | 0 | 0 | 2.981853 | 2.070389 | 3.292782 |
| ILMN_277 | Rbp2 | 0.000424 | 0.208598 | 3 | 3 | 0 | 2.471198 | 2.471198 | Higher in W | 0 | 0 | 0 | 2.847997 | 2.678072 | 1.887525 |
| ILMN_290 | Arfgap1 | 0.000459 | 0.214078 | 3 | 3 | 0.282666 | 3.719991 | 3.437325 | Higher in W | 0 | 0.847997 | 0 | 3.655352 | 4.50462 | 3 |
| ILMN_122 | Thada | 0.00046 | 0.214078 | 3 | 3 | 2.856172 | 0 | 2.856172 | Higher in N | 2.104337 | 3.017922 | 3.446256 | 0 | 0 | 0 |
| ILMN_267 | Slc17a4 | 0.000467 | 0.214463 | 3 | 3 | 0 | 2.517577 | 2.517577 | Higher in W | 0 | 0 | 0 | 2.608809 | 3.017922 | 1.925999 |
| ILMN_316 | Olfr685 | 0.000486 | 0.216699 | 3 | 3 | 2.396842 | 0 | 2.396842 | Higher in N | 2.104337 | 2.981853 | 2.104337 | 0 | 0 | 0 |
| ILMN_271 | Cartpt | 0.000506 | 0.217945 | 3 | 3 | 0.792837 | 6.897942 | 6.105105 | Higher in W | 0 | 0 | 2.378512 | 5.791814 | 6.744161 | 8.157852 |
| ILMN_123 | Aqr | 0.000509 | 0.217945 | 3 | 3 | 3.444808 | 0.379168 | 3.065641 | Higher in N | 3.678072 | 3.711495 | 2.944858 | 0 | 0 | 1.137504 |
| ILMN_122 | 1700084C | 0.000521 | 0.220236 | 3 | 3 | 0 | 2.490829 | 2.490829 | Higher in W | 0 | 0 | 0 | 3 | 1.887525 | 2.584963 |
| ILMN_259 | Ak8 | 0.000531 | 0.220811 | 3 | 3 | 0 | 3.151624 | 3.151624 | Higher in W | 0 | 0 | 0 | 3.944858 | 2.292782 | 3.217231 |
| ILMN_275 | Rax | 0.000554 | 0.222716 | 3 | 3 | 0 | 2.546039 | 2.546039 | Higher in W | 0 | 0 | 0 | 2.350497 | 3.217231 | 2.070389 |
| ILMN_125 | Lamb1 | 0.000556 | 0.222716 | 3 | 3 | 2.932121 | 0.255178 | 2.676943 | Higher in N | 2.378512 | 3.247928 | 3.169925 | 0.765535 | 0 | 0 |
| ILMN_125 | Tmem229a | 0.000594 | 0.229062 | 3 | 3 | 0.226024 | 2.820706 | 2.594682 | Higher in W | 0 | 0 | 0.678072 | 3.217231 | 2.981853 | 2.263034 |
| ILMN_125 | Etl4 | 0.000604 | 0.229062 | 3 | 3 | 0.379168 | 3.105269 | 2.726101 | Higher in W | 0 | 0 | 1.137504 | 2.867896 | 3.169925 | 3.277985 |
| ILMN_242 | Zan | 0.000609 | 0.229062 | 3 | 3 | 3.726246 | 0.723308 | 3.002938 | Higher in N | 4.217231 | 3.364572 | 3.596935 | 1.321928 | 0 | 0.847997 |
| ILMN_312 | Cartpt | 0.000629 | 0.229062 | 3 | 3 | 1.56444 | 6.467029 | 4.902589 | Higher in W | 1.722466 | 0.678072 | 2.292782 | 5.361066 | 6.223036 | 7.816984 |
| ILMN_242 | Capns2 | 0.000641 | 0.229062 | 3 | 3 | 0 | 4.368241 | 4.368241 | Higher in W | 0 | 0 | 0 | 5.442943 | 4.754888 | 2.906891 |
| ILMN_125 | Hif1an | 0.000641 | 0.229062 | 3 | 3 | 0 | 2.473225 | 2.473225 | Higher in W | 0 | 0 | 0 | 3.035624 | 2.536053 | 1.847997 |
| ILMN_272 | Ccr8 | 0.000645 | 0.229062 | 3 | 3 | 0 | 2.520926 | 2.520926 | Higher in W | 0 | 0 | 0 | 2.887525 | 1.807355 | 2.867896 |
| ILMN_264 | Leap2 | 0.000698 | 0.242934 | 3 | 3 | 2.859395 | 0.175356 | 2.684038 | Higher in N | 3.185867 | 2.070389 | 3.321928 | 0.263034 | 0.263034 | 0 |
| ILMN_243 | Six4 | 0.000722 | 0.243781 | 3 | 3 | 3.129167 | 0.605192 | 2.523975 | Higher in N | 2.807355 | 3.201634 | 3.378512 | 0.678072 | 0 | 1.137504 |
| ILMN_124 | 1110004F | 0.000723 | 0.243781 | 3 | 3 | 3.156634 | 0.255178 | 2.901456 | Higher in N | 3.292782 | 2.432959 | 3.744161 | 0 | 0.765535 | 0 |
| ILMN_125 | Odf3b | 0.00075 | 0.245059 | 3 | 3 | 0 | 2.595153 | 2.595153 | Higher in W | 0 | 0 | 0 | 2.350497 | 3.364572 | 2.070389 |
| ILMN_254 | Sec62 | 0.000763 | 0.245059 | 3 | 3 | 0.045835 | 3.013645 | 2.96781 | Higher in W | 0 | 0 | 0.137504 | 2.459432 | 3.972693 | 2.608809 |
| ILMN_122 | Dtna | 0.000767 | 0.245059 | 3 | 3 | 0 | 2.391775 | 2.391775 | Higher in W | 0 | 0 | 0 | 1.722466 | 2.867896 | 2.584963 |
| ILMN_263 | Mfng | 0.000867 | 0.256709 | 3 | 3 | 3.005044 | 0.087678 | 2.917365 | Higher in N | 2.070389 | 3.277985 | 3.666757 | 0 | 0 | 0.263034 |
| ILMN_122 | Esrrb | 0.000908 | 0.266338 | 3 | 3 | 0 | 3.422645 | 3.422645 | Higher in W | 0 | 0 | 0 | 3.104337 | 4.602884 | 2.560715 |
| ILMN_259 | Satb2 | 0.000951 | 0.27417 | 3 | 3 | 2.685094 | 0 | 2.685094 | Higher in N | 1.807355 | 3.247928 | 3 | 0 | 0 | 0 |
| ILMN_276 | Ugt3a1 | 0.000962 | 0.274626 | 3 | 3 | 0.400545 | 3.049577 | 2.649032 | Higher in W | 0 | 0 | 1.201634 | 3.321928 | 3.104337 | 2.722466 |
| ILMN_300 | Lpar3 | 0.000992 | 0.278065 | 3 | 3 | 3.16365 | 0 | 3.16365 | Higher in N | 3.754888 | 3.70044 | 2.035624 | 0 | 0 | 0 |
| ILMN_122 | Olfr705 | 0.001007 | 0.279728 | 3 | 3 | 2.47932 | 0.045835 | 2.433486 | Higher in N | 3.087463 | 2.584963 | 1.765535 | 0 | 0.137504 | 0 |

| | | | | | | | | | | | | | | | |
|---|---|---|---|---|---|---|---|---|---|---|---|---|---|---|---|
| ILMN_265 | Apc | 0.001067 | 0.291364 | 3 | 3 | 0 | 2.660016 | 2.660016 | Higher in V | 0 | 0 | 0 | 2.981853 | 3.232661 | 1.765535 |
| ILMN_268 | Adamts20 | 0.001076 | 0.291364 | 3 | 3 | 0.585674 | 3.41408 | 2.828406 | Higher in V | 1.378512 | 0 | 0.378512 | 2.867896 | 3.608809 | 3.765535 |
| ILMN_265 | Wdr77 | 0.001131 | 0.300997 | 3 | 3 | 2.844038 | 0 | 2.844038 | Higher in N | 3.185867 | 3.498251 | 1.847997 | 0 | 0 | 0 |
| ILMN_123 | Lef1 | 0.001181 | 0.305251 | 3 | 3 | 2.679517 | 0 | 2.679517 | Higher in N | 2.405992 | 2.035624 | 3.596935 | 0 | 0 | 0 |
| ILMN_122 | Wdr81 | 0.001195 | 0.305251 | 3 | 3 | 0.226024 | 3.122875 | 2.896852 | Higher in V | 0 | 0 | 0.678072 | 3.277985 | 3.857981 | 2.232661 |
| ILMN_121 | Ctbp1 | 0.001233 | 0.311492 | 3 | 3 | 0 | 2.906121 | 2.906121 | Higher in V | 0 | 0 | 0 | 3.321928 | 3.548437 | 1.847997 |
| ILMN_259 | Hadh | 0.001239 | 0.311492 | 3 | 3 | 0.045835 | 2.382656 | 2.336822 | Higher in V | 0.137504 | 0 | 0 | 1.632268 | 2.906891 | 2.608809 |
| ILMN_281 | Olfr99 | 0.001363 | 0.323625 | 3 | 3 | 0.333333 | 2.719791 | 2.386457 | Higher in V | 0 | 0 | 1 | 2.232661 | 2.944858 | 2.981853 |
| ILMN_122 | Aph1c | 0.00137 | 0.323625 | 3 | 3 | 2.577002 | 0.194988 | 2.382015 | Higher in N | 1.887525 | 3.121015 | 2.722466 | 0 | 0 | 0.584963 |
| ILMN_264 | Gm711 | 0.001397 | 0.323625 | 3 | 3 | 4.09641 | 1.445333 | 2.651077 | Higher in N | 4.285402 | 3.786596 | 4.217231 | 0.847997 | 2.350497 | 1.137504 |
| ILMN_254 | Eri2 | 0.001502 | 0.342216 | 3 | 3 | 4.38229 | 0.723308 | 3.658981 | Higher in N | 4.649615 | 4.452859 | 4.044394 | 0 | 0 | 2.169925 |
| ILMN_273 | Gm4477 // | 0.001534 | 0.344704 | 3 | 3 | 0.733878 | 4.295513 | 3.561635 | Higher in V | 1 | 1.201634 | 0 | 4.995485 | 4.837943 | 3.053111 |
| ILMN_276 | Dio2 | 0.001617 | 0.358298 | 3 | 3 | 2.582697 | 0 | 2.582697 | Higher in N | 2.867896 | 3.247928 | 1.632268 | 0 | 0 | 0 |
| ILMN_268 | Fign | 0.00167 | 0.365743 | 3 | 3 | 0 | 2.453654 | 2.453654 | Higher in V | 0 | 0 | 0 | 2.070389 | 1.925999 | 3.364572 |
| ILMN_276 | Cbfa2t3 | 0.00168 | 0.365743 | 3 | 3 | 0 | 2.61837 | 2.61837 | Higher in V | 0 | 0 | 0 | 2.944858 | 3.277985 | 1.632268 |
| ILMN_124 | Rdh12 | 0.00169 | 0.365743 | 3 | 3 | 0.559357 | 3.557522 | 2.998165 | Higher in V | 0 | 1.678072 | 0 | 3.336283 | 4 | 3.336283 |
| ILMN_276 | Ppp2r4 | 0.001707 | 0.365743 | 3 | 3 | 2.797306 | 0 | 2.797306 | Higher in N | 3.121015 | 1.722466 | 3.548437 | 0 | 0 | 0 |
| ILMN_122 | 2310034C | 0.001709 | 0.365743 | 3 | 3 | 0.440643 | 3.528899 | 3.088256 | Higher in V | 0 | 0 | 1.321928 | 4.405992 | 3.217231 | 2.963474 |
| ILMN_300 | Raver2 | 0.001918 | 0.396276 | 3 | 3 | 0.733878 | 4.253361 | 3.519483 | Higher in V | 0 | 0 | 2.201634 | 4.285402 | 4.529821 | 3.944858 |
| ILMN_126 | Pikfyve | 0.001925 | 0.396276 | 3 | 3 | 0 | 2.73035 | 2.73035 | Higher in V | 0 | 0 | 0 | 1.722466 | 2.847997 | 3.620586 |
| ILMN_121 | Rasl12 | 0.001947 | 0.396276 | 3 | 3 | 0.126171 | 2.554178 | 2.428007 | Higher in V | 0 | 0.378512 | 0 | 1.765535 | 2.560715 | 3.336283 |
| ILMN_248 | Tubgcp5 | 0.001947 | 0.396276 | 3 | 3 | 3.342575 | 0.605192 | 2.737383 | Higher in N | 3.350497 | 3.57289 | 3.104337 | 1.678072 | 0 | 0.137504 |
| ILMN_306 | Slc12a2 | 0.001964 | 0.396276 | 3 | 3 | 3.438806 | 0.777808 | 2.660998 | Higher in N | 4.044394 | 3.485427 | 2.786596 | 0 | 1.263034 | 1.070389 |
| ILMN_274 | Slc38a9 | 0.001997 | 0.397887 | 3 | 3 | 0 | 2.43927 | 2.43927 | Higher in V | 0 | 0 | 0 | 2.944858 | 1.485427 | 2.887525 |
| ILMN_124 | Arhgef2 | 0.002014 | 0.39847 | 3 | 3 | 0.67619 | 3.228871 | 2.552681 | Higher in V | 0.765535 | 0 | 1.263034 | 3.121015 | 2.678072 | 3.887525 |
| ILMN_124 | Clps | 0.002039 | 0.39847 | 3 | 3 | 2.621602 | 0.194988 | 2.426614 | Higher in N | 3.392317 | 2.584963 | 1.887525 | 0.584963 | 0 | 0 |
| ILMN_268 | Ret | 0.002059 | 0.39847 | 3 | 3 | 2.665576 | 0 | 2.665576 | Higher in N | 3.104337 | 3.307429 | 1.584963 | 0 | 0 | 0 |
| ILMN_269 | Dpep2 | 0.002153 | 0.409409 | 3 | 3 | 4.043174 | 0.602452 | 3.440722 | Higher in N | 3.307429 | 4.867896 | 3.954196 | 0 | 0 | 1.807355 |
| ILMN_122 | Loxhd1 | 0.002159 | 0.409409 | 3 | 3 | 0 | 2.430065 | 2.430065 | Higher in V | 0 | 0 | 0 | 3.307429 | 1.632268 | 2.350497 |
| ILMN_122 | Ston2 | 0.002171 | 0.409409 | 3 | 3 | 0 | 2.323833 | 2.323833 | Higher in V | 0 | 0 | 0 | 3.053111 | 2.432959 | 1.485427 |
| ILMN_252 | Fam81b | 0.002206 | 0.413489 | 3 | 3 | 2.99984 | 0.379168 | 2.620672 | Higher in N | 3.307429 | 3.459432 | 2.232661 | 1.137504 | 0 | 0 |
| ILMN_265 | Gucd1 | 0.002382 | 0.433322 | 3 | 3 | 0 | 2.651337 | 2.651337 | Higher in V | 0 | 0 | 0 | 3.620586 | 1.678072 | 2.655352 |
| ILMN_297 | Il22ra1 | 0.00247 | 0.442082 | 3 | 3 | 0.642 | 3.132915 | 2.490915 | Higher in V | 0.925999 | 0 | 1 | 3.432959 | 3.643856 | 2.321928 |
| ILMN_281 | Ercc6l2 | 0.002503 | 0.442839 | 3 | 3 | 2.455977 | 0.087678 | 2.368299 | Higher in N | 1.536053 | 2.678072 | 3.153805 | 0 | 0 | 0.263034 |
| ILMN_275 | Psma8 | 0.00255 | 0.446338 | 3 | 3 | 3.222459 | 0.512018 | 2.710442 | Higher in N | 3.797013 | 2.963474 | 2.906891 | 0 | 1.536053 | 0 |
| ILMN_272 | 1700029J | 0.002588 | 0.448055 | 3 | 3 | 0.792837 | 3.617813 | 2.824976 | Higher in V | 0 | 2 | 0.378512 | 3.620586 | 3.446256 | 3.786596 |
| ILMN_310 | Ccdc93 | 0.002636 | 0.453851 | 3 | 3 | 3.348984 | 0.512018 | 2.836967 | Higher in N | 2.70044 | 3.954196 | 3.392317 | 1.536053 | 0 | 0 |
| ILMN_244 | Vmn2r89 | 0.002694 | 0.458801 | 3 | 3 | 2.768961 | 0.308666 | 2.460294 | Higher in N | 3.536053 | 2.70044 | 2.070389 | 0 | 0.925999 | 0 |
| ILMN_254 | Il20rb | 0.003076 | 0.494432 | 3 | 3 | 0.733878 | 3.887242 | 3.153364 | Higher in V | 2.201634 | 0 | 0 | 3.857981 | 3.733354 | 4.070389 |
| ILMN_121 | Arid5b | 0.003087 | 0.494432 | 3 | 3 | 3.43433 | 0.992243 | 2.442087 | Higher in N | 3.560715 | 3.405992 | 3.336283 | 0.263034 | 0.678072 | 2.035624 |
| ILMN_245 | Tpcn1 | 0.003112 | 0.494432 | 3 | 3 | 0.226024 | 3.218451 | 2.992427 | Higher in V | 0 | 0 | 0.678072 | 4.485427 | 2.906891 | 2.263034 |
| ILMN_277 | Aldh8a1 | 0.003122 | 0.494432 | 3 | 3 | 4.18224 | 1.306129 | 2.876111 | Higher in N | 4.078951 | 4.061776 | 4.405992 | 2.070389 | 0 | 1.847997 |
| ILMN_121 | Rnf24 | 0.003264 | 0.512883 | 3 | 3 | 2.378228 | 0 | 2.378228 | Higher in N | 2.867896 | 2.944858 | 1.321928 | 0 | 0 | 0 |
| ILMN_271 | Il1r1 | 0.003443 | 0.537245 | 3 | 3 | 0 | 3.216945 | 3.216945 | Higher in V | 0 | 0 | 0 | 1.722466 | 4.343408 | 3.584963 |
| ILMN_125 | Jakmip2 | 0.0035 | 0.542489 | 3 | 3 | 0 | 3.279395 | 3.279395 | Higher in V | 0 | 0 | 0 | 4.371559 | 3.744161 | 1.722466 |
| ILMN_267 | Cers6 | 0.003545 | 0.542489 | 3 | 3 | 2.55672 | 0 | 2.55672 | Higher in N | 1.925999 | 3.744161 | 2 | 0 | 0 | 0 |
| ILMN_302 | Ssxb2 | 0.003545 | 0.542489 | 3 | 3 | 3.425927 | 0.972795 | 2.453132 | Higher in N | 3.666757 | 3.087463 | 3.523562 | 0 | 1.847997 | 1.070389 |
| ILMN_271 | Bsn | 0.003635 | 0.548284 | 3 | 3 | 0.512018 | 3.178749 | 2.666731 | Higher in V | 0 | 0 | 1.536053 | 2.906891 | 3.906891 | 2.722466 |
| ILMN_123 | A530032D | 0.003749 | 0.552046 | 3 | 3 | 3.260377 | 0.255178 | 3.005198 | Higher in N | 3.857981 | 4.035624 | 1.887525 | 0 | 0 | 0.765535 |
| ILMN_280 | Defb29 | 0.003752 | 0.552046 | 3 | 3 | 1.118715 | 3.60923 | 2.490515 | Higher in V | 1.678072 | 0 | 1.678072 | 3.350497 | 3.916477 | 3.560715 |
| ILMN_125 | Tex13a | 0.00382 | 0.553943 | 3 | 3 | 2.390215 | 0 | 2.390215 | Higher in N | 2.201634 | 1.536053 | 3.432959 | 0 | 0 | 0 |
| ILMN_279 | Pmp2 | 0.003823 | 0.553943 | 3 | 3 | 0 | 2.380021 | 2.380021 | Higher in V | 0 | 0 | 0 | 2.632268 | 1.321928 | 3.185867 |
| ILMN_316 | Olfr194 /// | 0.004118 | 0.582421 | 3 | 3 | 3.334444 | 0.687834 | 2.64661 | Higher in N | 3.536053 | 3.485427 | 2.981853 | 0.137504 | 0 | 1.925999 |
| ILMN_122 | Paqr5 | 0.004388 | 0.615088 | 3 | 3 | 0.544089 | 3.289957 | 2.745868 | Higher in V | 0 | 1.632268 | 0 | 2.678072 | 3.104337 | 4.087463 |
| ILMN_242 | Slc12a6 | 0.004519 | 0.620363 | 3 | 3 | 0 | 2.517992 | 2.517992 | Higher in V | 0 | 0 | 0 | 1.432959 | 3.584963 | 2.536053 |
| ILMN_125 | Gabrb3 | 0.004548 | 0.621051 | 3 | 3 | 3.137142 | 0.194988 | 2.942155 | Higher in N | 4.485427 | 2.925999 | 2 | 0 | 0.584963 | 0 |
| ILMN_264 | Smyd1 | 0.004893 | 0.648506 | 3 | 3 | 0.356796 | 3.669323 | 3.312527 | Higher in V | 0 | 0 | 1.070389 | 4.50462 | 2.070389 | 4.432959 |
| ILMN_255 | Tst | 0.004936 | 0.649924 | 3 | 3 | 0.61999 | 2.985507 | 2.365517 | Higher in V | 0.137504 | 0 | 1.722466 | 3.201634 | 2.584963 | 3.169925 |
| ILMN_284 | Slc47a1 | 0.004987 | 0.652842 | 3 | 3 | 5.652779 | 1.005974 | 4.646805 | Higher in N | 7.158862 | 5.232661 | 4.566815 | 0 | 3.017922 | 0 |
| ILMN_258 | Etfdh | 0.005037 | 0.653939 | 3 | 3 | 0 | 2.693441 | 2.693441 | Higher in V | 0 | 0 | 0 | 2.608809 | 1.536053 | 3.93546 |
| ILMN_260 | Rnf139 | 0.005109 | 0.657795 | 3 | 3 | 0 | 2.589159 | 2.589159 | Higher in V | 0 | 0 | 0 | 1.887525 | 3.916477 | 1.963474 |
| ILMN_296 | Ypel4 | 0.005187 | 0.657918 | 3 | 3 | 3.237656 | 0.421011 | 2.816644 | Higher in N | 4.224966 | 2.137504 | 3.350497 | 0 | 1.263034 | 0 |
| ILMN_125 | Gtf2ird1 | 0.005255 | 0.657918 | 3 | 3 | 0 | 2.554809 | 2.554809 | Higher in V | 0 | 0 | 0 | 1.263034 | 3.419539 | 2.981853 |
| ILMN_263 | Il1rapl1 | 0.005263 | 0.657918 | 3 | 3 | 2.992713 | 0.512018 | 2.480695 | Higher in N | 3.620586 | 2.321928 | 3.035624 | 0 | 1.536053 | 0 |
| ILMN_313 | Prdm8 | 0.005275 | 0.657918 | 3 | 3 | 2.523488 | 0 | 2.523488 | Higher in N | 3.678072 | 1.432959 | 2.459432 | 0 | 0 | 0 |
| ILMN_125 | Astn2 | 0.005321 | 0.658183 | 3 | 3 | 0.629175 | 3.095941 | 2.466766 | Higher in V | 0 | 0 | 1.887525 | 3.104337 | 2.981853 | 3.201634 |
| ILMN_254 | Gm14692 | 0.00544 | 0.663853 | 3 | 3 | 2.717749 | 0.087678 | 2.630071 | Higher in N | 4.053111 | 2.292782 | 1.807355 | 0 | 0 | 0.263034 |
| ILMN_259 | Snap91 | 0.00559 | 0.670761 | 3 | 3 | 0.226024 | 2.92966 | 2.703636 | Higher in V | 0.678072 | 0 | 0 | 1.807355 | 4.153805 | 2.827819 |
| ILMN_267 | Cebpz | 0.005751 | 0.681314 | 3 | 3 | 0.559357 | 2.91328 | 2.353923 | Higher in V | 0 | 0 | 1.678072 | 3.121015 | 3.185867 | 2.432959 |
| ILMN_275 | Il34 | 0.005906 | 0.692077 | 3 | 3 | 3.874826 | 0.828476 | 3.04635 | Higher in N | 3.776104 | 4.061776 | 3.786596 | 0 | 0 | 2.485427 |
| ILMN_305 | Smagp | 0.00608 | 0.702379 | 3 | 3 | 0.574155 | 3.190498 | 2.616343 | Higher in V | 1.722466 | 0 | 0 | 3.632268 | 2.378512 | 3.560715 |
| ILMN_124 | Abl1 | 0.006162 | 0.703092 | 3 | 3 | 3.06815 | 0.512018 | 2.556132 | Higher in N | 2.201634 | 3.754888 | 3.247928 | 0 | 1.536053 | 0 |
| ILMN_123 | Mrap | 0.006283 | 0.703379 | 3 | 3 | 3.457578 | 1.080613 | 2.376965 | Higher in N | 3.121015 | 3.666757 | 3.584963 | 0 | 2.104337 | 1.137504 |
| ILMN_122 | Frmd5 | 0.006342 | 0.703379 | 3 | 3 | 0.087678 | 2.480052 | 2.392374 | Higher in V | 0 | 0 | 0.263034 | 1.847997 | 3.744161 | 1.847997 |
| ILMN_315 | Ramp3 | 0.006346 | 0.703379 | 3 | 3 | 4.7283 | 2.052994 | 2.675306 | Higher in N | 4.596935 | 4.887525 | 4.70044 | 1.536053 | 3.485427 | 1.137504 |
| ILMN_313 | Thnsl1 | 0.00635 | 0.703379 | 3 | 3 | 0.559357 | 3.002623 | 2.443265 | Higher in V | 0 | 1.678072 | 0 | 3.378512 | 3.307429 | 2.321928 |
| ILMN_274 | Pigt | 0.006597 | 0.720215 | 3 | 3 | 2.740086 | 0 | 2.740086 | Higher in N | 3.916477 | 1.321928 | 2.981853 | 0 | 0 | 0 |
| ILMN_269 | Pkd1l2 | 0.006736 | 0.724632 | 3 | 3 | 2.364351 | 0 | 2.364351 | Higher in N | 1.201634 | 2.485427 | 3.405992 | 0 | 0 | 0 |
| ILMN_262 | Rassf2 | 0.006804 | 0.724632 | 3 | 3 | 2.822807 | 0 | 2.822807 | Higher in N | 4.008989 | 3.137504 | 1.321928 | 0 | 0 | 0 |
| ILMN_125 | Mettl24 | 0.006845 | 0.724632 | 3 | 3 | 1.516146 | 4.313297 | 2.797151 | Higher in V | 2.378512 | 0 | 2.169925 | 3.972693 | 4.638074 | 4.329124 |
| ILMN_297 | Rnf43 | 0.006935 | 0.724632 | 3 | 3 | 1.045835 | 3.413332 | 2.367497 | Higher in V | 2 | 1.137504 | 0 | 3.153805 | 3.104337 | 3.981853 |
| ILMN_123 | 2010005H | 0.00698 | 0.724933 | 3 | 3 | 0.544089 | 3.06179 | 2.517701 | Higher in V | 0 | 0 | 1.632268 | 3.035624 | 3.827819 | 2.321928 |
| ILMN_123 | Samd14 | 0.007219 | 0.735293 | 3 | 3 | 2.806198 | 0.356796 | 2.449402 | Higher in N | 3.765535 | 2.887525 | 1.765535 | 1.070389 | 0 | 0 |

| | | | | | | | | | | | | | | | |
|---|---|---|---|---|---|---|---|---|---|---|---|---|---|---|---|
| ILMN_124 | Mapk12 | 0.00726 | 0.737085 | 3 | 3 | 3.378988 | 0.723308 | 2.655679 | Higher in N | 3.053111 | 3.733354 | 3.350497 | 0 | 0 | 2.169925 |
| ILMN_264 | Igf1 | 0.007286 | 0.737367 | 3 | 3 | 2.978669 | 0.282666 | 2.696004 | Higher in N | 1.722466 | 4.177918 | 3.035624 | 0 | 0.847997 | 0 |
| ILMN_260 | Cntln | 0.00756 | 0.757826 | 3 | 3 | 0 | 2.880111 | 2.880111 | Higher in W | 0 | 0 | 0 | 1.263034 | 4.0268 | 3.350497 |
| ILMN_261 | Mmp10 | 0.007632 | 0.760172 | 3 | 3 | 6.628017 | 3.641805 | 2.986212 | Higher in N | 7.778734 | 6.249825 | 5.855491 | 4.61471 | 2.432959 | 3.877744 |
| ILMN_124 | Calr3 | 0.007648 | 0.760172 | 3 | 3 | 0.615999 | 3.005018 | 2.389019 | Higher in W | 1.847997 | 0 | 0 | 2.560715 | 3.472488 | 2.981853 |
| ILMN_303 | Slc7a2 | 0.007672 | 0.760172 | 3 | 3 | 2.55102 | 0 | 2.55102 | Higher in N | 4 | 1.765535 | 1.887525 | 0 | 0 | 0 |
| ILMN_267 | Lyg1 | 0.007792 | 0.764184 | 3 | 3 | 0.226024 | 5.45518 | 5.229156 | Higher in W | 0 | 0 | 0.678072 | 8.224966 | 5.375039 | 2.765535 |
| ILMN_259 | Krt6b | 0.008073 | 0.772199 | 3 | 3 | 0 | 4.993839 | 4.993839 | Higher in W | 0 | 0 | 0 | 7.429616 | 5.381975 | 2.169925 |
| ILMN_267 | Ciita | 0.008127 | 0.773137 | 3 | 3 | 1.354501 | 4.596424 | 3.241923 | Higher in W | 0.925999 | 0 | 3.137504 | 4.711495 | 4.981853 | 4.095924 |
| ILMN_123 | Hbp1 | 0.008355 | 0.781637 | 3 | 3 | 0 | 2.474302 | 2.474302 | Higher in W | 0 | 0 | 0 | 2.70044 | 3.584963 | 1.137504 |
| ILMN_279 | Cdsn | 0.008602 | 0.794466 | 3 | 3 | 2.790659 | 7.28694 | 4.496281 | Higher in W | 0.765535 | 4.343408 | 3.263034 | 9.056367 | 6.716991 | 6.087463 |
| ILMN_287 | Srp19 | 0.008681 | 0.799408 | 3 | 3 | 0.69013 | 3.318736 | 2.628606 | Higher in W | 0 | 2.070389 | 0 | 3.432959 | 3.867896 | 2.655352 |
| ILMN_121 | Slc39a4 | 0.009019 | 0.817369 | 3 | 3 | 3.933527 | 0.801997 | 3.13153 | Higher in N | 2.925999 | 4.649615 | 4.224966 | 0 | 2.405992 | 0 |
| ILMN_270 | Dennd5b | 0.009302 | 0.825369 | 3 | 3 | 1.202936 | 4.210901 | 3.007964 | Higher in W | 2.608809 | 1 | 0 | 4.578939 | 4.867896 | 3.185867 |
| ILMN_277 | Comp | 0.009359 | 0.828184 | 3 | 3 | 2.837659 | 5.504659 | 2.667 | Higher in W | 1.584963 | 3.620586 | 3.307429 | 4.584963 | 6.29094 | 5.638074 |
| ILMN_297 | Rpap2 | 0.009453 | 0.831622 | 3 | 3 | 3.982156 | 0.907489 | 3.074667 | Higher in N | 4.459432 | 3.963474 | 3.523562 | 0 | 0 | 2.722466 |
| ILMN_290 | Coro2b | 0.009625 | 0.839965 | 3 | 3 | 3.152295 | 0.712501 | 2.439794 | Higher in N | 2.827819 | 3.336283 | 3.292782 | 0 | 0 | 2.137504 |
| ILMN_316 | Mrpl1 | 0.009737 | 0.842876 | 3 | 3 | 2.901744 | 0.559357 | 2.342387 | Higher in N | 2.350497 | 3.722466 | 2.632268 | 1.678072 | 0 | 0 |
| ILMN_248 | Cacna1a | 0.009825 | 0.843466 | 3 | 3 | 1.224519 | 3.682684 | 2.458165 | Higher in W | 0 | 2.137504 | 1.536053 | 3 | 4.314697 | 3.733354 |
| ILMN_263 | Rtbdn | 0.010006 | 0.852109 | 3 | 3 | 2.499667 | 0.045835 | 2.453832 | Higher in N | 2.867896 | 3.560715 | 1.070389 | 0 | 0.137504 | 0 |
| ILMN_273 | Kif12 | 0.010141 | 0.854372 | 3 | 3 | 0.495142 | 3.110873 | 2.615731 | Higher in W | 0 | 0 | 1.485427 | 2.847997 | 4.314697 | 2.169925 |
| ILMN_306 | Tbx3 | 0.010384 | 0.864954 | 3 | 3 | 3.038261 | 0.642 | 2.396261 | Higher in N | 3.722466 | 2.807355 | 2.584963 | 0 | 0 | 1.925999 |
| ILMN_277 | Map3k5 | 0.01089 | 0.884479 | 3 | 3 | 0.459504 | 2.953075 | 2.493571 | Higher in W | 1.378512 | 0 | 0 | 2.104337 | 2.584963 | 4.169925 |
| ILMN_123 | Rbfox1 | 0.010998 | 0.886985 | 3 | 3 | 0.716853 | 3.318695 | 2.601842 | Higher in W | 1.887525 | 0 | 0.263034 | 2.584963 | 4.426265 | 2.944858 |
| ILMN_262 | Dhcr24 | 0.011095 | 0.886985 | 3 | 3 | 0.819811 | 3.658503 | 2.838692 | Higher in W | 2.459432 | 0 | 0 | 3.972693 | 2.906891 | 4.095924 |
| ILMN_125 | Ly6g6c | 0.011154 | 0.886985 | 3 | 3 | 5.047794 | 7.768838 | 2.721043 | Higher in W | 4.121015 | 5.70044 | 5.321928 | 9.175175 | 7.320124 | 6.811214 |
| ILMN_125 | Tmem229a | 0.011158 | 0.886985 | 3 | 3 | 1.269118 | 3.681249 | 2.41213 | Higher in W | 0 | 2.321928 | 1.485427 | 3.232661 | 4.300124 | 3.510962 |
| ILMN_250 | Wnt3 | 0.011228 | 0.886985 | 3 | 3 | 0 | 2.738915 | 2.738915 | Higher in W | 0 | 0 | 0 | 3.643856 | 3.57289 | 1 |
| ILMN_285 | 4930502E1 | 0.011484 | 0.88767 | 3 | 3 | 3.958209 | 0.845351 | 3.112858 | Higher in N | 3.93546 | 4.921246 | 3.017922 | 2.536053 | 0 | 0 |
| ILMN_121 | Glrx3 | 0.011493 | 0.88767 | 3 | 3 | 3.56935 | 0.773976 | 2.795374 | Higher in N | 3.620586 | 2.765535 | 4.321928 | 0 | 0 | 2.321928 |
| ILMN_249 | Flrt1 | 0.011612 | 0.88767 | 3 | 3 | 1.063011 | 3.530018 | 2.467007 | Higher in W | 0 | 1.263034 | 1.925999 | 4.017922 | 4.1127 | 2.459432 |
| ILMN_257 | Plekha1 | 0.011711 | 0.88767 | 3 | 3 | 1.143428 | 3.887553 | 2.744125 | Higher in W | 0 | 1.137504 | 2.292782 | 4.053111 | 2.807355 | 4.802193 |
| ILMN_253 | Gpr113 | 0.012112 | 0.88767 | 3 | 3 | 2.861434 | 0.194988 | 2.666446 | Higher in N | 3.485427 | 1.201634 | 3.89724 | 0 | 0 | 0.584963 |
| ILMN_289 | 2310002J1 | 0.012714 | 0.88767 | 3 | 3 | 0.990285 | 4.246188 | 3.255904 | Higher in W | 1.485427 | 0 | 1.485427 | 5.820179 | 4.432959 | 2.485427 |
| ILMN_272 | Krtap21-1 | 0.013138 | 0.88767 | 3 | 3 | 0 | 4.533235 | 4.533235 | Higher in W | 0 | 0 | 0 | 7.090642 | 4.786596 | 1.722466 |
| ILMN_252 | Extl2 | 0.013296 | 0.88767 | 3 | 3 | 3.285475 | 0.528321 | 2.757154 | Higher in N | 4.364572 | 1.847997 | 3.643856 | 0 | 0 | 1.584963 |
| ILMN_244 | Zfp287 | 0.013697 | 0.88767 | 3 | 3 | 3.318899 | 0.856821 | 2.462078 | Higher in N | 3.70044 | 3.070389 | 3.185867 | 0 | 0.137504 | 2.432959 |
| ILMN_270 | Sp6 | 0.013796 | 0.88767 | 3 | 3 | 2.024 | 5.609205 | 3.585205 | Higher in W | 2.906891 | 2.786596 | 0.378512 | 7.119979 | 5.744161 | 3.963474 |
| ILMN_316 | Akr1c19 | 0.013864 | 0.88767 | 3 | 3 | 0.69013 | 3.102362 | 2.412233 | Higher in W | 0 | 2.070389 | 0 | 3.364572 | 3.620586 | 2.321928 |
| ILMN_254 | 5430419D | 0.013931 | 0.88767 | 3 | 3 | 0 | 2.549488 | 2.549488 | Higher in W | 0 | 0 | 0 | 1.722466 | 4.247928 | 1.678072 |
| ILMN_273 | Chrna1 | 0.014019 | 0.88767 | 3 | 3 | 0.754345 | 3.117177 | 2.362832 | Higher in W | 2.263034 | 0 | 0 | 2.722466 | 3.336283 | 3.292782 |
| ILMN_124 | Olfr1085 | 0.014074 | 0.88767 | 3 | 3 | 3.465934 | 0.792837 | 2.673097 | Higher in N | 4 | 2.632268 | 3.765535 | 0 | 2.378512 | 0 |
| ILMN_267 | Tmem70 | 0.015052 | 0.88767 | 3 | 3 | 0 | 2.32991 | 2.32991 | Higher in W | 0 | 0 | 0 | 3.153805 | 3.070389 | 0.765535 |
| ILMN_310 | Masp2 | 0.015573 | 0.88767 | 3 | 3 | 0.836987 | 3.352902 | 2.515914 | Higher in W | 0 | 0 | 2.510962 | 3.733354 | 3.017922 | 3.307429 |
| ILMN_267 | Olfr536 | 0.015609 | 0.88767 | 3 | 3 | 2.385749 | 0 | 2.385749 | Higher in N | 0.847997 | 3.608809 | 2.70044 | 0 | 0 | 0 |
| ILMN_279 | Cd59b | 0.015669 | 0.88767 | 3 | 3 | 3.846461 | 1.249129 | 2.597332 | Higher in N | 3.93546 | 3.776104 | 3.827819 | 2.981853 | 0 | 0.765535 |
| ILMN_124 | Bdnf | 0.015707 | 0.88767 | 3 | 3 | 3.02873 | 0.615999 | 2.412731 | Higher in N | 4 | 2.981853 | 2.104337 | 0 | 1.847997 | 0 |
| ILMN_124 | Pp2d1 | 0.015921 | 0.88767 | 3 | 3 | 3.376876 | 0.792837 | 2.584039 | Higher in N | 2.744161 | 4.153805 | 3.232661 | 2.378512 | 0 | 0 |
| ILMN_247 | Arsi | 0.016025 | 0.88767 | 3 | 3 | 1.373119 | 5.296596 | 3.923478 | Higher in W | 0 | 1.137504 | 2.981853 | 7.141596 | 5.426265 | 3.321928 |
| ILMN_122 | Ube2c | 0.016394 | 0.88767 | 3 | 3 | 0.308666 | 2.735915 | 2.427249 | Higher in W | 0 | 0.925999 | 0 | 2.867896 | 4.017922 | 1.321928 |
| ILMN_244 | Wdr31 | 0.016543 | 0.88767 | 3 | 3 | 4.09698 | 1.56496 | 2.53202 | Higher in N | 3.906891 | 4.705978 | 3.678072 | 2.807355 | 0 | 1.887525 |
| ILMN_244 | Mtif3 | 0.016783 | 0.88767 | 3 | 3 | 0 | 3.163759 | 3.163759 | Higher in W | 0 | 0 | 0 | 2.765535 | 5.292782 | 1.432959 |
| ILMN_276 | Sohlh2 | 0.017221 | 0.88767 | 3 | 3 | 0 | 2.690988 | 2.690988 | Higher in W | 0 | 0 | 0 | 1 | 4.307429 | 2.765535 |
| ILMN_283 | Glis3 | 0.01732 | 0.88767 | 3 | 3 | 3.028928 | 0.701446 | 2.327482 | Higher in N | 2.560715 | 3.847997 | 2.678072 | 2.104337 | 0 | 0 |
| ILMN_125 | Clcnka | 0.017896 | 0.88767 | 3 | 3 | 4.401573 | 1.092662 | 3.308912 | Higher in N | 3.744161 | 5.416164 | 4.044394 | 0 | 3.277985 | 0 |
| ILMN_262 | Rbpms2 | 0.017897 | 0.88767 | 3 | 3 | 3.152494 | 0.810986 | 2.341508 | Higher in N | 3.446256 | 3.104337 | 2.906891 | 0 | 0 | 2.432959 |
| ILMN_122 | Taar3 | 0.018354 | 0.88767 | 3 | 3 | 3.175896 | 0.801997 | 2.373899 | Higher in N | 3.536053 | 2.655352 | 3.336283 | 2.405992 | 0 | 0 |
| ILMN_297 | N4bp2 | 0.018857 | 0.88767 | 3 | 3 | 3.210877 | 5.574292 | 2.363415 | Higher in W | 3.754888 | 4.292782 | 1.584963 | 5.590961 | 5.880196 | 5.251719 |
| ILMN_277 | Habp4 | 0.019315 | 0.88767 | 3 | 3 | 3.592122 | 0.877423 | 2.7147 | Higher in N | 3.857981 | 4.240314 | 2.678072 | 0 | 0 | 2.632268 |
| ILMN_264 | Eif4e3 | 0.019447 | 0.88767 | 3 | 3 | 4.567583 | 1.178684 | 3.388899 | Higher in N | 3.944858 | 5.472488 | 4.285402 | 0 | 0 | 3.536053 |
| ILMN_122 | E230008N | 0.019886 | 0.88767 | 3 | 3 | 2.626944 | 0.194988 | 2.431957 | Higher in N | 2.485427 | 1.201634 | 4.193772 | 0.584963 | 0 | 0 |
| ILMN_261 | Ctdp1 | 0.021054 | 0.88767 | 3 | 3 | 1.143874 | 3.835442 | 2.691568 | Higher in W | 0 | 0.378512 | 3.053111 | 3.217231 | 4.201634 | 4.087463 |
| ILMN_259 | Vmp1 | 0.021332 | 0.88767 | 3 | 3 | 4.075741 | 1.429207 | 2.646534 | Higher in N | 3.523562 | 4.590961 | 4.1127 | 1.070389 | 0 | 3.217231 |
| ILMN_280 | Gbp10 | 0.021476 | 0.88767 | 3 | 3 | 3.281012 | 0.845351 | 2.435661 | Higher in N | 2.744161 | 3.201634 | 3.89724 | 0 | 0 | 2.536053 |
| ILMN_295 | Myt1l | 0.02162 | 0.88767 | 3 | 3 | 3.722342 | 1.005974 | 2.716368 | Higher in N | 3.981853 | 3.877744 | 3.307429 | 0 | 0 | 3.017922 |
| ILMN_262 | Krt31 | 0.021697 | 0.88767 | 3 | 3 | 0 | 4.548894 | 4.548894 | Higher in W | 0 | 0 | 0 | 7.231701 | 5.213347 | 1.201634 |
| ILMN_124 | Hook1 | 0.021908 | 0.88767 | 3 | 3 | 2.824325 | 0.495142 | 2.329183 | Higher in N | 3.807355 | 1.432959 | 3.232661 | 0 | 1.485427 | 0 |
| ILMN_123 | Nampt | 0.021914 | 0.88767 | 3 | 3 | 0 | 2.729145 | 2.729145 | Higher in W | 0 | 0 | 0 | 0.925999 | 4.517276 | 2.744161 |
| ILMN_269 | Tgm5 | 0.022729 | 0.88767 | 3 | 3 | 0.987825 | 3.791922 | 2.804098 | Higher in W | 0 | 2.963474 | 0 | 4.209453 | 4.240314 | 2.925999 |
| ILMN_316 | Olfr243 | 0.023302 | 0.88767 | 3 | 3 | 3.224059 | 0.877423 | 2.346637 | Higher in N | 2.925999 | 3.498251 | 3.247928 | 0 | 0 | 2.632268 |
| ILMN_121 | Drd4 | 0.023984 | 0.88767 | 3 | 3 | 3.863723 | 1.495357 | 2.368366 | Higher in N | 3.446256 | 3.93546 | 4.209453 | 1.432959 | 0 | 3.053111 |
| ILMN_271 | Slitrk4 | 0.024019 | 0.88767 | 3 | 3 | 2.381383 | 5.688044 | 3.306661 | Higher in W | 3.523562 | 0 | 3.620586 | 6.467606 | 4.661065 | 5.93546 |
| ILMN_121 | Klk1b8 | 0.024229 | 0.88767 | 3 | 3 | 4.618018 | 2.232248 | 2.38577 | Higher in N | 5.569856 | 3.837943 | 4.446256 | 2.350497 | 0.847997 | 3.498251 |
| ILMN_296 | Tbkbp1 | 0.024848 | 0.88767 | 3 | 3 | 4.373373 | 1.634989 | 2.738384 | Higher in N | 4.078951 | 3.827819 | 5.213347 | 0 | 1.485427 | 3.419539 |
| ILMN_271 | Nup133 | 0.025215 | 0.88767 | 3 | 3 | 1.143428 | 4.090353 | 2.946925 | Higher in W | 2.944858 | 0.485427 | 0 | 3.017922 | 5.498251 | 3.754888 |
| ILMN_282 | Cybrd1 | 0.025653 | 0.88767 | 3 | 3 | 3.450166 | 0.900147 | 2.550019 | Higher in N | 4.350497 | 3 | 3 | 0 | 0 | 2.70044 |
| ILMN_124 | Krt24 | 0.026298 | 0.88767 | 3 | 3 | 3.721788 | 1.029154 | 2.692634 | Higher in N | 3.655352 | 4.292782 | 3.217231 | 0 | 0 | 3.087463 |
| ILMN_123 | Krtap9-3 | 0.02652 | 0.88767 | 3 | 3 | 0.853572 | 5.165148 | 4.311576 | Higher in W | 0 | 2.560715 | 0 | 7.767522 | 5.321928 | 2.405992 |
| ILMN_124 | Itgae | 0.026972 | 0.88767 | 3 | 3 | 0 | 2.519029 | 2.519029 | Higher in W | 0 | 0 | 0 | 4.255501 | 2.536053 | 0.765535 |
| ILMN_300 | Fhad1 | 0.026998 | 0.88767 | 3 | 3 | 0.962508 | 3.429549 | 2.467041 | Higher in W | 2.887525 | 0 | 0 | 3.053111 | 3.459432 | 3.776104 |
| ILMN_266 | Rptn | 0.027862 | 0.88767 | 3 | 3 | 1.121524 | 6.265605 | 5.144081 | Higher in W | 0 | 3.364572 | 0 | 9.470252 | 6.205549 | 3.121015 |
| ILMN_275 | Mllt1 | 0.027886 | 0.88767 | 3 | 3 | 3.815336 | 1.487569 | 2.327767 | Higher in N | 2.807355 | 4.232661 | 4.405992 | 1.807355 | 0 | 2.655352 |
| ILMN_279 | Il12rb1 | 0.02789 | 0.88767 | 3 | 3 | 2.52288 | 0 | 2.52288 | Higher in N | 3.584963 | 0.485427 | 3.498251 | 0 | 0 | 0 |

| | | | | | | | | | | | | | | | |
|---|---|---|---|---|---|---|---|---|---|---|---|---|---|---|---|
| ILMN_251 | Ppp2r2b | 0.027969 | 0.88767 | 3 | 3 | 0.968964 | 3.614064 | 2.645101 | Higher in W | 0 | 0 | 2.906891 | 2.744161 | 4.321928 | 3.776104 |
| ILMN_122 | Apaf1 | 0.028334 | 0.88767 | 3 | 3 | 1.295517 | 3.806798 | 2.511282 | Higher in W | 0.765535 | 0 | 3.121015 | 3.053111 | 3.981853 | 4.385431 |
| ILMN_277 | Klf16 | 0.028795 | 0.88767 | 3 | 3 | 1.58257 | 4.078456 | 2.495886 | Higher in W | 0.925999 | 0.485427 | 3.336283 | 3.169925 | 4.177918 | 4.887525 |
| ILMN_261 | Lce3f /// LC | 0.028917 | 0.88767 | 3 | 3 | 0.764261 | 3.384038 | 2.619778 | Higher in W | 0 | 2.292782 | 0 | 4.711495 | 3.336283 | 2.104337 |
| ILMN_258 | Cpeb2 | 0.029125 | 0.88767 | 3 | 3 | 2.125939 | 4.871455 | 2.745515 | Higher in W | 0 | 2.944858 | 3.432959 | 5.266787 | 5.193772 | 4.153805 |
| ILMN_122 | Arhgef15 | 0.030183 | 0.88767 | 3 | 3 | 0.981619 | 3.49939 | 2.517771 | Higher in W | 0 | 0 | 2.944858 | 3.336283 | 4.161888 | 3 |
| ILMN_124 | Sprr4 | 0.030305 | 0.88767 | 3 | 3 | 0 | 3.473916 | 3.473916 | Higher in W | 0 | 0 | 0 | 6.179909 | 1.137504 | 3.104337 |
| ILMN_259 | Kcnn2 | 0.03046 | 0.88767 | 3 | 3 | 3.887369 | 1.107309 | 2.78006 | Higher in N | 4.566815 | 3.405992 | 3.689299 | 0 | 3.321928 | 0 |
| ILMN_123 | Olfr371 | 0.030763 | 0.88767 | 3 | 3 | 3.389139 | 0.845351 | 2.543788 | Higher in N | 2.321928 | 4.523562 | 3.321928 | 0 | 2.536053 | 0 |
| ILMN_285 | Defb6 | 0.03086 | 0.88767 | 3 | 3 | 1.155325 | 4.151275 | 2.99595 | Higher in W | 0.765535 | 2.70044 | 0 | 6.098032 | 3.169925 | 3.185867 |
| ILMN_264 | Rprm | 0.031798 | 0.88767 | 3 | 3 | 1.665162 | 5.734722 | 4.06956 | Higher in W | 4.857981 | 0.137504 | 0 | 7.088523 | 5.495056 | 4.620586 |
| ILMN_122 | Pan3 | 0.032153 | 0.88767 | 3 | 3 | 0.861654 | 3.29263 | 2.430976 | Higher in W | 0 | 0 | 2.584963 | 3.137504 | 4.307429 | 2.432959 |
| ILMN_125 | Sp6 | 0.033468 | 0.88767 | 3 | 3 | 2.675637 | 6.05946 | 3.383823 | Higher in W | 2.169925 | 4.655352 | 1.201634 | 7.608809 | 6.507795 | 4.061776 |
| ILMN_124 | Pkn3 | 0.033593 | 0.88767 | 3 | 3 | 0.981619 | 3.601215 | 2.619596 | Higher in W | 0 | 0 | 2.944858 | 3.666757 | 4.50462 | 2.632268 |
| ILMN_263 | Psmc6 | 0.03382 | 0.88767 | 3 | 3 | 1.913822 | 4.308471 | 2.394649 | Higher in W | 0 | 3.419539 | 2.321928 | 4.255501 | 4.590961 | 4.078951 |
| ILMN_125 | Cdsn | 0.034218 | 0.88767 | 3 | 3 | 2.174312 | 4.883975 | 2.709663 | Higher in W | 3.596935 | 2.925999 | 0 | 5.649615 | 4.529821 | 4.472488 |
| ILMN_251 | Zfp36l2 | 0.034427 | 0.88767 | 3 | 3 | 3.814977 | 1.005974 | 2.809003 | Higher in N | 5.044394 | 2.678072 | 3.722466 | 3.017922 | 0 | 0 |
| ILMN_121 | Amigo1 | 0.03469 | 0.88767 | 3 | 3 | 1.760319 | 4.389818 | 2.6295 | Higher in W | 0 | 1.432959 | 3.847997 | 4.643856 | 4.472488 | 4.053111 |
| ILMN_308 | Whrn | 0.034869 | 0.88767 | 3 | 3 | 0.993951 | 3.468557 | 2.474606 | Higher in W | 0 | 0 | 2.981853 | 4.224966 | 2.963474 | 3.217231 |
| ILMN_122 | Wee1 | 0.034901 | 0.88767 | 3 | 3 | 0.819811 | 3.157496 | 2.337685 | Higher in W | 2.459432 | 0 | 0 | 2.887525 | 4.263034 | 2.321928 |
| ILMN_274 | Prkd1 | 0.036816 | 0.88767 | 3 | 3 | 3.473098 | 1.040338 | 2.43276 | Higher in N | 3.510962 | 3.070389 | 3.837943 | 0 | 0 | 3.121015 |
| ILMN_253 | Tmem45a | 0.037873 | 0.88767 | 3 | 3 | 3.984684 | 1.206862 | 2.777821 | Higher in N | 4.185867 | 3.446256 | 4.321928 | 3.620586 | 0 | 0 |
| ILMN_297 | LOC10050 | 0.038743 | 0.88767 | 3 | 3 | 1.07241 | 3.503854 | 2.431444 | Higher in W | 0 | 3.217231 | 0 | 3.485427 | 3.733354 | 3.292782 |
| ILMN_125 | Elovl4 | 0.040111 | 0.88767 | 3 | 3 | 1.605192 | 5.306444 | 3.701252 | Higher in W | 0 | 4.137504 | 0.678072 | 7.369815 | 5.013462 | 3.536053 |
| ILMN_122 | Fkbp6 | 0.041943 | 0.88767 | 3 | 3 | 3.452151 | 0.975333 | 2.476818 | Higher in N | 2.584963 | 4.523562 | 3.247928 | 0 | 0 | 2.925999 |
| ILMN_121 | Cdkn2c | 0.042359 | 0.88767 | 3 | 3 | 1.092662 | 4.091148 | 2.998486 | Higher in W | 0 | 3.277985 | 0 | 4.385431 | 5.402586 | 2.485427 |
| ILMN_123 | Krt6b | 0.042866 | 0.88767 | 3 | 3 | 0.379168 | 2.907064 | 2.527896 | Higher in W | 1.137504 | 0 | 0 | 4.958843 | 1.201634 | 2.560715 |
| ILMN_295 | Kremen2 | 0.043823 | 0.88767 | 3 | 3 | 1.545251 | 4.116335 | 2.571084 | Higher in W | 0 | 1.137504 | 3.498251 | 5.213347 | 3.981853 | 3.153805 |
| ILMN_277 | Tgm3 | 0.044359 | 0.88767 | 3 | 3 | 0 | 2.397687 | 2.397687 | Higher in W | 0 | 0 | 0 | 4.329124 | 2.378512 | 0.485427 |
| ILMN_121 | Mmab | 0.045253 | 0.88767 | 3 | 3 | 4.207507 | 1.846903 | 2.360604 | Higher in N | 4.57289 | 4.201634 | 3.847997 | 1.807355 | 0 | 3.733354 |
| ILMN_297 | Sprr1a | 0.047491 | 0.88767 | 3 | 3 | 3.349318 | 5.789654 | 2.440336 | Higher in W | 3.827819 | 2.827819 | 3.392317 | 7.667466 | 5.904484 | 3.797013 |
| ILMN_124 | Kprp | 0.047551 | 0.88767 | 3 | 3 | 0 | 3.818432 | 3.818432 | Higher in W | 0 | 0 | 0 | 6.464342 | 4.72792 | 0.263034 |
| ILMN_259 | Tet1 | 0.047607 | 0.88767 | 3 | 3 | 0.810986 | 3.537088 | 2.726102 | Higher in W | 0 | 0 | 2.432959 | 4.857981 | 1.536053 | 4.217231 |
| ILMN_122 | Casp12 | 0.048029 | 0.88767 | 3 | 3 | 1.045835 | 3.473098 | 2.427264 | Higher in W | 0 | 0 | 3.137504 | 4.070389 | 2.510962 | 3.837943 |
| ILMN_122 | Agt | 0.048224 | 0.88767 | 3 | 3 | 1.413438 | 4.467237 | 3.053799 | Higher in W | 0 | 4.240314 | 0 | 4.596935 | 5.104337 | 3.70044 |
| ILMN_296 | Rufy3 | 0.049684 | 0.88767 | 3 | 3 | 1.051268 | 3.374224 | 2.322956 | Higher in W | 0 | 3.153805 | 0 | 2.744161 | 3.378512 | 4 |
